# Investigating the effect of e-cigarette use on quitting smoking in adults aged 25 years or more using the PATH study

**DOI:** 10.12688/f1000research.26167.1

**Published:** 2020-09-07

**Authors:** Peter N. Lee, John S. Fry

**Affiliations:** 1 P.N.Lee Statistics and Computing, Sutton, Surrey, SM2 5DA, UK; 2 RoeLee Statistics Ltd, Sutton, Surrey, SM2 5DA, UK

**Keywords:** Cigarettes, Confounding, Over-adjustment, E-cigarettes, Cessation, Modelling

## Abstract

**Background:** Part of the evidence on harms and benefits of e-cigarettes concerns whether using e-cigarettes encourages smokers to quit.  With limited results from controlled trials, and weaknesses in much epidemiological data, we addressed this using nationally representative prospective study data, with detailed accounting for factors associated with quitting.

**Methods:** Analyses used data for adults aged 25+ years from Waves 1 to 3 of the US PATH study. Separate analyses concerned follow-up from Waves 1 to 2, 2 to 3 and 1 to 3.  The main analyses related baseline ever e-cigarette use (or e-product use at Wave 2) to having quit at follow-up, adjusting for predictors of quitting derived from 55 candidates.  Sensitivity analyses omitted adults who had never used other products, linked quitting to current rather than ever e-cigarette use, used modified values of some predictors using later recorded data, or (in Wave 1 to 3 analysis only) also adjusted for quitting by Wave 2.

**Results:** In the main analyses, unadjusted odds ratios (ORs) of quitting for ever e-cigarette use were 1.29 (95% CI 1.01-1.66), 1.52 (1.26-1.83) and 1.47 (1.19-1.82) for the Wave 1 to 2, 2 to 3, and 1 to 3 analyses.  These estimates reduced after adjustment, to 1.23 (0.94-1.61), 1.51 (1.24-1.85) and 1.39 (1.11-1.74).  The final models, including between six and nine predictors, always included household income, everyday/someday smoking, wanting to smoke after waking and having tried quitting, with other variables included in specific analyses.  Quitting rates remained elevated in e-cigarette users in all sensitivity analyses. ORs were increased where other product users were omitted.  Adjusted ORs of quitting for current e-cigarette use were 1.41 (1.06-1.89), 1.30 (1.01-1.67) and 1.56 (1.21-2.00).

**Conclusions:** The results suggest e-cigarettes may assist adult smokers to quit, particularly in individuals not using other nicotine products, and who are current e-cigarette users.

## Introduction

Although it is believed that e-cigarettes cause far less harm to their users than do cigarettes ( Nutt *et al.*, 2014), the introduction of e-cigarettes may theoretically have various adverse and beneficial effects ( Lee *et al.*, 2019). Adverse effects would occur if the use of e-cigarettes encouraged initiation of smoking, if smokers intending to quit take up e-cigarettes instead, or if smokers take up e-cigarettes without reducing their cigarette consumption. Beneficial effects would occur if individuals who would otherwise have continued cigarette smoking switch instead to e-cigarette use, if simultaneous use of e-cigarettes helps smokers to materially reduce their cigarette consumption, or if use of e-cigarettes helps established smokers to quit. Here we present results relating to the last of these possibilities, the effect of e-cigarette use on quitting.

Information on e-cigarette use as an aid to quitting comes from various sources. Evidence from randomised controlled trials comparing smokers assigned a nicotine e-cigarette or a placebo ( Baldassarri *et al.*, 2018; Bullen *et al.*, 2013; Caponnetto *et al.*, 2013; Caponnetto *et al.*, 2019; Masiero *et al.*, 2019), comparing e-cigarettes with nicotine replacement therapy ( Hajek *et al.*, 2019; Li *et al.*, 2020) or comparing e-cigarettes with nicotine patches ( Walker *et al.*, 2020) generally indicates higher quit rates in the nicotine e-cigarette group, but this is not always the case ( Halpern *et al.*, 2018). A non-randomised study in which smokers were offered free e-cigarettes ( Hajek *et al.*, 2015) also found that those who accepted them were more likely to quit. While such evidence avoids uncontrolled confounding, it can be argued that such trials do not fully reflect what happens in the general population, where smokers choose to try or not try e-cigarettes without being allocated them. 

Evidence that smoking rates have declined in the US and UK over a period where e-cigarette use has been increasing ( Beard *et al.*, 2020; West *et al.*, 2016b; Zhu *et al.*, 2017) is suggestive of a beneficial effect of e-cigarette use on quitting, but is limited by the difficulty of taking account of other factors affecting smoking rates.

Epidemiological studies are an alternative approach, but while most of such studies show a positive relationship between e-cigarettes and smoking cessation, a recent review considered that the evidence is inconclusive due to the low quality of the research ( Malas *et al.*, 2016). Problems involve the use of cross-sectional studies, the use of unrepresentative populations, failure to limit attention to established e-cigarette users, and the failure fully to take into account the many factors associated with quitting smoking. An expert reaction ( West *et al.*, 2016a) made clear that a meta-analysis perversely claiming that e-cigarette use was associated with a reduced risk of quitting ( Kalkhoran & Glantz, 2016) suffered from such weaknesses. Restricting attention to cohort studies (other than the study we analyse here), which determine e-cigarette use at baseline and quitting at follow-up, it is clear that by now there are quite a number of studies that report somewhat higher quit rates in those using e-cigarettes, (e.g. ( Jackson *et al.*, 2019; Mantey *et al.*, 2017; Piper *et al.*, 2019; Snow *et al.*, 2018; Weaver *et al.*, 2018; Young-Wolff *et al.*, 2018; Zhuang *et al.*, 2016), and though there are also many that did not find any clear association, (e.g. ( Bowler *et al.*, 2017; Brose *et al.*, 2015; Chiang *et al.*, 2019; Comiford *et al.*, 2020; Flacco *et al.*, 2019; Grana *et al.*, 2014; Harrington *et al.*, 2015; Lozano *et al.*, 2019; Pasquereau *et al.*, 2017; Sweet *et al.*, 2019; Wang *et al.*, 2017; Wu *et al.*, 2018), it is rare to find a study ( Al-Delaimy *et al.*, 2015) suggesting that e-cigarettes inhibits quitting.

Here we describe results from a prospective study aimed at avoiding such weaknesses. It is based on the Population Assessment of Tobacco and Health (PATH) study, a nationally representative cohort study of tobacco use and how it affects the health of people in the US. Wave 1 was conducted from 12 September 2013 to 15 December 2014, and our analyses are based on data for this Wave and from two annual follow-ups (Waves 2 and 3). The data files made publicly available include extensive information on use of various types of tobacco products and on numerous variables linked to initiation of tobacco. In order to avoid complexities caused by consideration of younger adults who may only recently have initiated cigarette smoking, possibly only on a temporary basis, attention is limited to adults aged 25 years or more, an age when initiation of cigarettes is less common.

## Methods

Separate sets of analyses have been conducted for three periods, from Wave 1 to Wave 2 (period 1), from Wave 2 to Wave 3 (period 2) and from Wave 1 to Wave 3 (period 3). The analyses are based on individuals with relevant data available at Waves 1, 2 and 3 on smoking and e-cigarette use, and take account of the Wave 1 person-based weights. All analyses are limited to individuals aged 25 years or over at baseline.

### Definition of smoking, e-cigarette use and predictor variables

A current cigarette smoker is a “current established cigarette user” defined as “has ever smoked a cigarette, has smoked more than 100 cigarettes in life time, and currently smokes every day or some days”, while a former cigarette smoker is a “former established cigarette user” defined as “has ever smoked a cigarette, has smoked more than 100 cigarettes in life time, and now does not smoke at all”. Those who are neither current nor former cigarette smokers are not considered in the analyses.

A current e-cigarette user is a “current established e-cigarette user” defined as “has ever used an e-cigarette, has used fairly regularly and uses every day or some days”, while a former e-cigarette user is a “former established e-cigarette user” defined as “has ever used an e-cigarette, has used fairly regularly, and currently does not use at all”. An ever e-cigarette user is either a current or former e-cigarette user. At Wave 2 those who smoked other e-products (such as e-cigars, e-pipes or e-hookahs) are also included, so the definition relates to e-product rather than e-cigarette use.

The main analysis for each period relates ever e-cigarette use at baseline to the probability of quitting, with adjustment for predictor variables measured at baseline. The predictor variables have been selected from a pre-defined list of candidates classified into eight groups: demographics (A); general aspects of smoking (B); aspects of smoking specifically related to quitting (C); smoking by family and friends (D); awareness of hazards of smoking (E); health status (F); alcohol and drugs (G); and others (H).

The specific predictor variables are listed in the Results section, with fuller details of their definition given in the *Extended data.*

Where the baseline of the period studied is Wave 1, the values of the predictor variables used are as recorded at Wave 1. Where it is Wave 2, the values of some variables are amended to take into account data from Wave 1, as described in the *Extended data*.

### Main analysis

For each period, the main analysis was conducted in seven stages, preliminary counts and six further steps, each involving weighted logistic regression analyses.

**Counts**     Restricted to individuals who were current cigarette smokers at baseline, a frequency table was prepared, separated by quitting during follow-up and e-cigarette use at baseline by each of the adjustment variables. Missing values are shown, to indicate variables with high levels of missing values requiring special consideration in analysis.

**Step 1**     This is conducted in eight parts, each part corresponding to a group of predictor variables (A to H). For each part, the regressions first relate each predictor variable individually to quitting, with stepwise forward multiple regressions then carried out, with the most significant predictor variable introduced first, then the next most significant, and so on, until no more variable can be added that is significant at p < 0.01.

**Step 2**     This is in three sections, each involving stepwise forward multiple regressions. The first section considers all the variables found to be significant in Step 1 from groups A, B and C, the second considers those significant from groups D, E and F, and the third those significant from groups G and H.

**Step 3**     A final stepwise regression considers all the predictor variables remaining as significant in step 2. This generates a final list of predictors to be considered when relating ever e-cigarette use to quitting.

Each analysis in steps 1 to 3 is restricted to those with non-missing data for all the predictor variables considered in the particular analysis.

The final three steps are then based on all individuals with data on all the predictor variables in the final list.

**Step 4**     An unadjusted analysis relates ever e-cigarette use to quitting.

**Step 5** Stepwise regression analyses are run, introducing the predictor variables in the final list first, and then adding ever e-cigarettes as a predictor.

**Step 6**     Stepwise regressions similar to those in step 5 are run, but introducing ever e-cigarette use first rather than last.

The main results produced by the regression analyses are the odds ratios (ORs) and 95% confidence intervals (CIs) relating to each predictor of interest, and the significance of introducing that predictor into the model.

### Sensitivity analyses

While the main analyses relates quitting cigarettes during follow-up to ever e-cigarette use at baseline and the predictor variables considered include use of nicotine products other than cigarettes or e-cigarettes, four sensitivity analyses (S1 to S4) were also conducted, which are intended to give additional information on how dependent the ORs derived from the main analyses are on exactly how they are conducted. These are modifications of the main analyses:

S1 restricts attention to individuals who have never used other nicotine products;

S2 links quitting to current (rather than ever) e-cigarette use at baseline;

S3 adjusts, where necessary, for variables which take account of data recorded at the end of follow-up rather than just at baseline; and

S4, which applies only to the analyses based on quitting between Waves 1 and 3, additionally adjusts for whether the individual had already quit by Wave 2.

In each of S1 to S4 the analyses run were as in steps 4 to 6 of the main analyses and used the final set of predictor variables derived for the period they related to.

### Missing values and other details

For most of the 55 predictor variables considered, there were relatively few missing values, and the regressions could be run excluding the individuals with missing values for the predictors considered without material loss of power. However, for two predictors, where there were about 8% of missing values, individuals with missing data were assigned average values. Thus, for household income in the past 12 months, where data were recorded in five increasing levels, individuals recorded as unknown were assigned an income in the third level, $25,000 to $49,999, while for poverty status, where data were recorded in three levels, <100%, 100–199% and 200+% of the poverty guideline, individuals recorded as unknown were assigned a status in the second level. For living with a regular smoker who smoked inside your home during childhood, where about 16% of individuals were classified as “not ascertained” rather than “yes” or “no”, this answer was included as a separate level, thus the predictor was treated in analysis as having three levels.

For some predictors with multiple levels, the regression analyses were based on a single trend variable. This was only appropriate where the predictor variable represented increasing (or decreasing) levels of a characteristic.

Generally, the analyses were based on predictors recorded at the baseline Wave. Where the baseline Wave was Wave 2, however, and data were not available at Wave 2, Wave 1 data were used if appropriate. Also, if the Wave 2 predictor related to ever having done something, particularly when the variable concerned action in the last 12 months, individuals were counted as ever having done so if this was reported at Wave 1 or 2.

Further details of the process are given in the *Extended data*.

### Software

Relevant data were transferred for analysis to a ROELEE database, and analysed using the ROELEE program (Release 59, Build 49). All these analyses could be run using the R-program, using the “lm” function including the “weights=” option for weighted linear regression, and for stepwise regression using the “step” function specifying “method=forward” and test=”F”.

## Results

### Main analysis

Table 1 shows the predictor variables used in the final regression analysis or excluded at various stages of the preliminary analyses.

**Table 1.** Predictor variables included in the final regression analysis (Y) or excluded at steps 1, 2 or 3 (X1, X2, X3).

	Levels of variable ^a^	Wave 1 to 2 quitting	Wave 2 to 3 quitting	Wave 1 to 3 quitting
Smokers at baseline		6,503	6,847	6,490
Quit by follow-up		655 (10.1%)	633 (9.2%)	901 (13.9%)
Demographics (A)				
Age range	5	Y	X3	X2
Gender	2	X1	X1	X1
Hispanic origin	2	X2	X2	X2
Race	3	X1	X1	X1
Census region	4	X1	X1	X1
Total household income	5T	Y	Y	Y
Poverty status	3T	X1	-	X1
Total number in the household	5T	X1	-	X1
Highest grade level of school completed	6T	X2	Y	X2
Currently enrolled in a degree program	2	X1	X1	X1
Current employment status	8	X1	X1	X1
Aspects of smoking – general (B)				
Age range started smoking cigarettes fairly regularly	6	X1	X1	X1
Current someday cigarette smokers	2	Y	Y	Y
Cigarettes per day	C	Y	X3	Y
Ever used other tobacco products	2	X1	X1	X1
Frequently crave tobacco product(s)	5T	X1	X1	X1
Usually wants to smoke/use tobacco right after waking	5T	Y	Y	Y
After not smoking for a while, need to smoke to avoid discomfort	5T	X1	X1	X1
Can only go a couple of hours without smoking/tobacco	5T	X1	X1	X1
Aspects of smoking – specifically related to quitting (C)				
Have tried to quit completely	2	Y	Y	Y
Would find it hard to stop smoking/tobacco for a while	5T	X2	X2	X2
Times stopped smoking for one day or more in past year	4T	X1	X1	X1
Ever used a nicotine patch, gum, inhaler, nasal spray, lozenge or pill	2	X1	X1	X1
Ever used Chantix, varenicline or bupropion (Wellbutrin, Zyban)	2	X1	X1	Y
Plans to quit smoking/using tobacco product(s) for good	2	X1	X1	X1
Smoking/using tobacco product(s) really helps me feel better if feeling down	5	X1	X1	X1
Extent disapproval of smoking from friends and family led to thinking about quitting in past year	3T	X1	-	X1
Smoking by family and friends (D)				
Rules about smoking a combustible tobacco	3T	Y	X3	Y
Anyone who lives with you now smoke cigarettes	2	X3	X1	X2
Most people I spend time with are tobacco users	5T	X3	X3	X2
Lived with regular smoker who smoked inside your home during childhood	3	X1	Y	X2
Awareness of hazards of smoking (E)				
How often have you seen a list of chemicals in tobacco products in last 12 months	5T	X1	X1	X1
How often noticed health warnings on cigarette packages in past 30 days	5T	X1	X1	X1
Overall opinion of tobacco	5T	Y	X3	X2
Perception of harmfulness of cigarettes to health	5T	X1	X1	Y
Health status (F)				
Saw a medical doctor in past 12 months	2	X2	X1	X1
Body mass index	C	Y	X3	X1
Self-perception of physical health	5T	X1	X2	X1
Self-perception of quality of life	5T	X3	X3	X3
Last time significant problems with:				
Feeling very trapped, lonely, sad, etc.	4T	X1	X1	X1
Sleep troubles	4T	X1	X1	X1
Feeling very anxious, nervous, tense, etc.	4T	X1	X1	X1
Becoming very distressed with something reminded of past	4T	X1	X1	X1
Alcohol and drugs (G)				
Ever used alcohol	2	X1	X1	X1
Last time used alcohol or other drugs weekly or more often	5T	X1	X1	X1
Days drank an alcoholic beverage in past 30 days	31T	X1	X1	X1
Ever used marijuana, hash, THC or grass	2	X1	X1	X1
Ever used unprescribed Ritalin or Adderall	2	X1	X1	X1
Ever used unprescribed painkillers, sedatives or tranquilizers	2	X1	X1	X1
Ever used cocaine or crack	2	X1	X1	X1
Ever used stimulants like methamphetamine or speed	2	X1	X1	X1
Ever used any other drugs like heroin, inhalants, solvents, hallucinogens	2	X1	X1	X1
Other (H)				
Hours spent watching TV on a typical day	5T	X1	X3	X3
How often uses the Internet	7T	X3	-	X3
Time a day on social media sites	5T	-	X1	-

^a^ C = continuous variable, T = treated as a linear trend variable in regressions

For the analyses based on Waves 1 and 2, for example, 54 predictors were considered, 11 in group A, 8 in B, 8 in C, 4 in D, 4 in E, 8 in F, 9 in G, and 2 in H. Of the 55 predictors listed in Table 1, there was one variable in group H with no Wave 1 data. Of the 54 predictors considered in the Wave 1 and 2 analyses, 37 were excluded at step 1, marked X1 in Table 1. A further 4 were excluded at step 2 (X2). This left 13 variables considered in step 3, of which 4 were excluded (X3), with 9 included in the final model (Y).

For the analyses based on Waves 2 and 3 there were data available for 51 predictors, with 45 excluded (34 X1, 3 X2 and 8 X3) and 6 included in the final model. For those based on Waves 1 and 3 there were data on 54 predictors, with 46 excluded (35 X1, 8 X2 and 3 X3) and 8 included in the final model. 

Table 2 summarizes the results of the main analysis. Each analysis was based on between 6,000 and 7,000 adults with the percentage quitting varying from 9.1% to 13.1%. The unadjusted gateway-out effect varied from 1.29 to 1.52 in the three analyses. Adjustment only slightly reduced the estimates, the fully adjusted ORs being 1.23 (95%CI 0.94-1.61) for Wave 1 to 2, 1.51 (1.24-1.85) for Wave 2 to 3, and 1.39 (1.11-1.74) for Wave 1 to 3.

**Table 2.** Effect of adjustment on the OR (95% CI) for the relationship of ever regular e cigarette use ^a^ to quitting.

	Wave 1 to 2 quitting	Wave 2 to 3 quitting	Wave 1 to 3 quitting
Number in baseline in final regression	6,241	6,758	6,315
Number quitting	581 (9.3%)	616 (9.1%)	829 (13.1%)
Unadjusted	1.29 (1.01-1.66)	1.52 (1.26-1.83)	1.47 (1.19-1.82)
Adjusted for four most important variables	1.17 (0.90-1.52)	1.54 (1.26-1.88)	1.43 (1.14-1.78)
Adjusted for all variables included in final list	1.23 (0.94-1.61) [9 variables]	1.51 (1.24-1.85) [6 variables]	1.39 (1.11-1.74) [8 variables]

^a^Where the baseline is Wave 1, the predictor is ever regular e-cigarette use, where it is Wave 2, it is ever regular e-product use

Table 3 shows the full models used, showing the effect estimates for each of the predictor variables used to adjust the relationship of ever regular e-cigarette use to quitting. Where the same adjustment variable was included in each model, the effect estimates were generally quite similar and always in the same direction. As regards aspects of smoking, smokers were found to be less likely to quit if they were everyday smokers, were more likely to smoke right after waking up, had not previously tried to quit, smoked more cigarettes per day, lived in a home with more relaxed rules about smoking, lived with a smoker in childhood, had a better opinion of tobacco, or had a lesser perception of cigarettes as harmful. Interestingly, they were also less likely to quit if they had ever used pharmaceutical aids for quitting, i.e. Chantix, varenicline or bupropion (Wellbutrin, Zyban). Smokers were also less likely to quit if they were worse off and worse educated. Higher age (particularly above age 74 years) and higher BMI were also associated with a greater likelihood to quit.

**Table 3.** ORs related to quitting cigarettes in the final models used in the main analyses.

	Wave 1 to 2 quitting	Wave 2 to 3 quitting	Wave 1 to 3 quitting
Ever regularly used e-cigarette = yes	1.23 (0.94-1.61)	1.51 (1.24-1.85)	1.39 (1.11-1.74)
Age range			
25–34	Reference	Not included	Not included
35–44	0.95 (0.74-1.21)		
45–54	0.85 (0.66-1.10)		
55–64	1.20 (0.92-1.57)		
65–74	1.42 (0.98-2.06)		
75+	3.31 (1.84-5.97)		
Total household income (per level increasing)	1.14 (1.05-1.23)	1.16 (1.07-1.25)	1.21 (1.13-1.29)
Current some day smoker = no	0.43 (0.34-0.54)	0.36 (0.30-0.44)	0.52 (0.43-0.64)
Highest grade or level at school completed (per level increasing)	Not included	1.10 (1.03-1.18)	Not included
Per cigarette per day	0.97 (0.96-0.99)	Not included	0.97 (0.96-0.98)
Usually wants to smoke/use tobacco right after waking up (per level increasing)	0.89 (0.83-0.95)	0.79 (0.74-0.84)	0.85 (0.81-0.90)
Have tried to quit completely = no	0.62 (0.51-0.75)	0.64 (0.53-0.77)	0.69 (0.58-0.82)
Ever used Chantix, varenicline or buproprion (Wellbutrin, Zyban)	Not included	Not included	0.78 (0.65-0.94)
Rules about smoking combustible tobacco at home (per level of decreasing stringency)	0.77 (0.68-0.88)	Not included	0.78 (0.70-0.87)
Lived with a regular smoker who smoked inside your home in childhood = no	Not included	1.41 (1.18-1.68)	Not included
Overall opinion of tobacco (per level of increasing negativity)	1.17 (1.07-1.29)	Not included	Not included
Perception of harmfulness of cigarettes to health (per level increasing)	Not included	Not included	1.13 (1.03-1.24)
Per unit of body mass index	1.02 (1.01-1.03)	Not included	Not included

### Sensitivity analyses

Table 4 summarises the results of the sensitivity analyses, showing the estimated ORs in each case from the fully adjusted analysis. The first line of results (“Main model”) repeats the estimates shown in Table 3.

**Table 4.** Comparing adjusted ORs for the effect of e-cigarette use on quitting in the main and sensitivity analyses.

	Wave 1 to 2 quitting		Wave 2 to 3 quitting		Wave 1 to 3 quitting	
	N(n)	OR (95% CI)	N(n)	OR (95% CI)	N(n)	OR (95% CI)
Main model	581 (78)	1.23 (0.94-1.61)	616 (160)	1.51 (1.24-1.85)	829 (118)	1.39 (1.11-1.74)
Sensitivity analysis 1 (Omitting ever users of other nicotine products)	148 (16)	2.04 (1.15-3.62)	122 (20)	1.69 (0.96-2.99)	217 (25)	2.22 (1.38-3.57)
Sensitivity analysis 2 (Linking quitting to current rather than ever e-cigarette use at baseline)	581 (64)	1.41 (1.06-1.89)	615 (89)	1.30 (1.01-1.67)	829 (95)	1.56 (1.21-2.00)
Sensitivity analysis 3 (Adjusting for variables taking account of data recorded at both baseline and end of follow-up)	655 (82)	1.20 (0.92-1.57)	591 (160)	1.66 (1.35-2.04)	901 (121)	1.43 (1.14-1.79)
Sensitivity analysis 4 (Adjust also for quitting by Wave 2)		Not applicable		Not applicable	443 (64)	1.43 (1.11-1.84)

N = total number quitting n = number of quitters among e-cigarette users (current users in sensitivity analysis 2, ever users otherwise)

Sensitivity analysis 1 excludes those who had ever used other nicotine products. The number of quitters is substantially reduced, as is the number using e-cigarettes (or e-products). However, the OR is increased, with ever regular users of e-cigarettes about twice as likely to quit cigarettes by the end of the follow-up period, though the confidence limits of the ORs are relatively wide.

In sensitivity analysis 2, quitting is linked to current rather than ever e-cigarette use. Here the ORs tend to be somewhat higher than in the main analysis (though not for the Wave 2 to 3 analysis).

The results for both sensitivity analyses 1 and 2 seem consistent with smokers being more likely to quit if, at baseline, e-cigarettes formed a more important part of the total tobacco use.

Sensitivity analysis 3 adjusts, where necessary, for variables which are modified to take account of data recorded at the end of follow-up, and not just at baseline, in an attempt to minimize “residual confounding”. The ORs were quite similar to those in the main analysis for Wave 1 to 2, or Wave 1 to 3 quitting, but were somewhat increased for Wave 2 to 3 quitting.

Sensitivity analysis 4, only applicable to the Wave 1 to 3 quitting analyses, adjusted also for having quit by Wave 2. This slightly increased the estimate from the main analysis.

### Discussion

This report summarizes evidence from Waves 1, 2 and 3 of the US PATH study relating to the possibility that e-cigarette use may increase the likelihood of smokers quitting cigarettes. All of the adjusted ORs estimated, which (as shown in Table 4) varied between 1.20 and 2.22, were consistent with this possibility, although not all the estimates were statistically significant at p < 0.05. Compared to the estimates from the main model, which related ever e-cigarette use at baseline to quitting by follow-up, ORs were increased (though based on far fewer quitters) when those who had ever used other products were omitted from the analysis. The ORs were also increased, in the analysis with Wave 1 as the baseline, when quitting was linked to current rather than ever e-cigarette use. In both the sensitivity analyses where the ORs were increased, e-cigarette use would have formed a greater proportion of current tobacco use at baseline.

Other related analyses based on the PATH study have previously been published, all of which are consistent with e-cigarette use increasing the probability of quitting cigarettes.

An analysis of 3,093 quit attempters based on adult data from Waves 1 and 2 ( Benmarhnia *et al.*, 2018) considered two endpoints – abstinence from smoking for at least 30 days and reduced cigarette consumption – and reported a significant increase in both endpoints related to using e-cigarettes to quit during the previous year, but no significant increase in either endpoint related to the use of approved pharmaceutical aids.

Another analysis based on Waves 1 and 2 ( Berry *et al.*, 2019), here limiting attention to adults aged 25 years or more, studied factors related to 30-day cigarette cessation and to at least a 50% reduction in cigarette consumption in multivariable logistic regression analyses, which included a number of the variables included as predictors in our analyses. While the model included e-cigarette use, this was defined not at baseline, but as new e-cigarette use at Wave 2. In this analysis large ORs were reported for everyday e-cigarette use both for cessation (7.88, 95% CI 4.45-13.95) and for 50% reduction in cigarette consumption (5.70, 3.47-9.35).

A further analysis based on Waves 1 and 2 ( Verplaetse *et al.*, 2019) considered adults aged 18+ years and reported that, compared to those who had never used e-cigarettes at Wave 1, quitting was increased in Wave 1 daily users (OR 1.56. 95%CI 1.12-2.18) but not in Wave 1 nondaily users (0.83, 0.68-1.02). Age, race and education were the only adjustment variables considered.

Analyses based on data from Waves 1, 2 and 3 ( Watkins *et al.*, 2020), conducted separately for adults aged 18–24 years and 25+ years, studied the relation of a variety of cessation strategies to short-term cessation (quit at Wave 2) and long-term cessation (quit at both Waves 2 and 3). Adjustments were made for a range of covariates. The authors reported that “substitution with e-cigarettes” did not predict long-term cessation but predicted short-term cessation for older daily smokers of 5 or more cigarettes a day.

An analysis based on data for adults from Waves 1, 2 and 3 ( Kalkhoran *et al.*, 2020), related current e-cigarette use at Wave 1 (defined as daily, non-daily or none) in cigarette smokers at Wave 1 to three cigarette abstinence endpoints: at Wave 2, at Wave 3 or at Waves 2 and 3 (prolonged abstinence). Adjustments were made for a fixed set of variables: age, sex, race/ethnicity, education, income, cigarettes per day, and having a first cigarette within 30 minutes of waking. Non-daily e-cigarette use was only associated with a small, non-significant increase in each of the abstinence endpoints, but daily e-cigarette use was associated with a clear increase in all three endpoints, with adjusted ORs of 1.53 (95%CI 1.04-2.23) for Wave 2 abstinence, 1.57 (1.12-2.21) for Wave 3 abstinence, and 1.77 (1.08-2.89). These results particularly seem quite similar to ours.

Strengths of our work include the use of a prospective study design based on a study population representative of the US, and analyses which take account of a very large number of other predictors of quitting, and restrict attention to established e-cigarette use.

Limitations relate to the relatively small number of quitters, leading to the decision not to study heterogeneity of the results by basic variables, such as sex, race or age group. Our decision to limit attention to those aged at least 25 years was based on the desire not to include young smokers whose smoking habits were not well established. We have also not studied persistent quitting, by relating Wave 1 e-cigarette use to quitting at both Waves 2 and 3. As our analyses were based on a pre-defined plan, and as data from Wave 4 are now available, we plan to address these issues in a further paper. This might, for example, relate Wave 1 e-cigarette use simultaneously to quitting at all subsequent waves, separating those who quit at all three waves, or at only one or two. 

For the present, our results clearly suggest that among adults aged 25 years or more, most of whom would not have initiated smoking recently, e-cigarettes may assist in helping smokers to quit, particularly if, at baseline, e-cigarettes form an important part of total tobacco use – i.e. for individuals who at baseline did not use products other than cigarettes or e-cigarettes, and who were current rather than ever e-cigarette users.

### Data availability

#### Underlying data

National Addiction & HIV Data Archive Program: Population Assessment of Tobacco and Health (PATH) Study [United States] Public-Use Files (ICPSR 36498), https://doi.org/10.3886/ICPSR36498.v8 ( United States Department of Health and Human Services (USDHHS), 2018).

The data are available under the Terms of Use as set out by ICPSR, which can be accessed when users start the process of downloading the data.

### Extended data

Open Science Framework: Investigating the effect of e-cigarette use on quitting smoking in adults aged 25 or more using the PATH study https://doi.org/10.17605/OSF.IO/F4WA7 ( Lee & Fry, 2020).

This project contains the following extended data file:

- Additional file_fuller details regarding the predictor variables used.docx

Data are available under the terms of the Creative Commons Zero “No rights reserved” data waiver (CC0 1.0 Public domain dedication).
