# Investigating the effect of e-cigarette use on quitting smoking in adults aged 25 years or more using the PATH study

**DOI:** 10.12688/f1000research.26167.2

**Published:** 2021-03-12

**Authors:** Peter N. Lee, John S. Fry

**Affiliations:** 1 P.N.Lee Statistics and Computing, Sutton, Surrey, SM2 5DA, UK; 2 RoeLee Statistics Ltd, Sutton, Surrey, SM2 5DA, UK

**Keywords:** Cigarettes, Confounding, Over-adjustment, E-cigarettes, Cessation, Modelling

## Abstract

**Background:** The evidence on harms and benefits of e-cigarettes partly concerns whether their use encourages smokers to quit.  We addressed this using data from the nationally representative PATH study, with detailed accounting for potential confounding variables.

**Methods:** We considered adults aged 25+.  Our original analyses, reported in version 1 of this paper, used data for Waves 1 to 3, separate analyses considering Waves 1 to 2, 2 to 3 and 1 to 3.  These related baseline ever e-cigarette use (or e-product use at Wave 2) to quitting at follow-up, adjusting for confounders derived from 55 candidates.  Sensitivity analyses omitted ever other product users, linked quitting to current e-cigarette use, and used values of some predictors modified using follow-up data.  Additional analyses used data for Waves 1 to 4, separately considering sustained, delayed and temporary quitting during Waves 1 to 3, 2 to 4 and 1 to 4.  Sensitivity analyses considered 30-day quitting, restricted attention to smokers attempting to quit, and considered ever smokeless tobacco or snus use.

**Results:** In the original analyses, unadjusted odds ratios (ORs) of quitting smoking forever e-cigarette use were 1.29 (95% CI 1.01-1.66), 1.52 (1.26-1.83) and 1.47 (1.19-1.82) for the Wave 1 to 2, 2 to 3, and 1 to 3 analyses.  These reduced after adjustment, to 1.23 (0.94-1.61), 1.51 (1.24-1.85) and 1.39 (1.11-1.74).  Quitting rates remained elevated in users in all sensitivity analyses.  The additional analyses found associations of e-cigarette use with sustained, delayed and temporary quitting, associations little affected by considering 30-day quitting, and only slightly reduced restricting attention to quit attempters.  Ever use of smokeless tobacco or snus also predicted increased quitting.

**Conclusions:** As does most evidence from clinical trials, other analyses of PATH, and other epidemiological studies, our results suggest using e-cigarettes helps adult smokers to quit.

## Abbreviations

CI, confidence interval; OR, odds ratio; PATH, Population Assessment of Tobacco and Health.

## Introduction

Although it is believed that e-cigarettes cause far less harm to their users than do cigarettes ( Nutt *et al*., 2014), the introduction of e-cigarettes may theoretically have various adverse and beneficial effects ( Lee *et al*., 2019). Adverse effects would occur if the use of e-cigarettes encouraged initiation of smoking, if smokers intending to quit take up e-cigarettes instead, or if smokers take up e-cigarettes without reducing their cigarette consumption. Beneficial effects would occur if individuals who would otherwise have continued cigarette smoking switch instead to e-cigarette use, if simultaneous use of e-cigarettes helps smokers to materially reduce their cigarette consumption, or if use of e-cigarettes helps established smokers to quit. Here we present results relating to the last of these possibilities, the effect of e-cigarette use on quitting.

Information on e-cigarette use as an aid to quitting comes from various sources. Evidence from randomised controlled trials comparing smokers assigned a nicotine e-cigarette or a placebo ( Baldassarri *et al*., 2018; Bullen *et al*., 2013; Caponnetto *et al*., 2013; Caponnetto *et al*., 2019; Eisenberg *et al*., 2020; Masiero *et al*., 2019), comparing e-cigarettes with NRT ( Hajek *et al*., 2019; Li *et al*., 2020) or comparing e-cigarettes with nicotine patches ( Walker *et al*., 2020) generally indicates higher quit rates in the nicotine e-cigarette group, although not all the differences cited were statistically significant (at p < 0.05), and one study did not find such higher quit rates ( Halpern *et al*., 2018). A non-randomised study in which smokers were offered free e-cigarettes ( Hajek *et al*., 2015) also found that those who accepted them were more likely to quit. That the evidence from trials suggests higher quit rates in those using e-cigarettes is also consistent with the findings of recent reviews ( Grabovac *et al*., 2020; Hartmann-Boyce *et al*., 2020; Zhang *et al*., 2021). While such evidence avoids uncontrolled confounding it can be argued that such trials do not fully reflect what happens in the general population, where smokers choose to try or not try e-cigarettes without being allocated them. 

Evidence that smoking rates have declined in the US and UK over a period where e-cigarette use has been increasing ( Beard *et al*., 2020; West *et al*., 2016b; Zhu *et al*., 2017) is suggestive of a beneficial effect of e-cigarette use on quitting, but is limited by the difficulty of taking account of other factors affecting smoking rates.

Epidemiological studies are an alternative approach, but while most of such studies show a positive relationship between e-cigarettes and smoking cessation, recent reviews have considered that the evidence is inconclusive due to the low quality of the research ( Malas *et al*., 2016; National Academies of Sciences Engineering and Medicine, 2018). Problems involve the use of cross-sectional studies, the use of unrepresentative populations, the use of non-comparable control groups, failure to limit attention to established e-cigarette users, and the failure fully to take into account the many factors associated with quitting smoking. An expert reaction ( West *et al*., 2016a) made clear that a meta-analysis perversely claiming that e-cigarette use was associated with a reduced risk of quitting ( Kalkhoran & Glantz, 2016) suffered from such weaknesses. Restricting attention to cohort studies (other than the study we analyse here) which determine e-cigarette use at baseline and quitting at follow-up, it is clear that by now there are quite a number of studies which report somewhat higher quit rates in those using e-cigarettes, (e.g. ( Mantey *et al*., 2017; Piper *et al*., 2019; Snow *et al*., 2018; Young-Wolff *et al*., 2018; Zhuang *et al*., 2016), and though there are also many that did not find any clear association, (e.g. ( Bowler *et al*., 2017; Brose *et al*., 2015; Chiang *et al*., 2019; Comiford *et al*., 2021; Flacco *et al*., 2019; Grana *et al*., 2014; Gravely *et al*., 2020; Harrington *et al*., 2015; Jackson *et al*., 2020; Lozano *et al*., 2019; Pasquereau *et al*., 2017; Sweet *et al*., 2019; Wang *et al*., 2017; Wu *et al*., 2018), it is rare to find studies ( Al Delaimy *et al*., 2015; Weaver *et al*., 2018) suggesting that e-cigarettes inhibits quitting.

Here we describe results from a prospective study aimed at avoiding such weaknesses. The main objective of our analyses is to quantify the relationship between e-cigarette use in smokers and subsequent cessation of smoking, with detailed adjustment for the multitude of factors that may differ between e-cigarette users and non-users. Our analyses are based on the Population Assessment of Tobacco and Health (PATH) study, a nationally representative cohort study of tobacco use and how it affects the health of people in the US. Wave 1 was conducted from 12 September 2013 to 15 December 2014, and our analyses are based on data for this Wave and from annual follow-ups. The data files made publicly available include extensive information on use of various types of tobacco products and on numerous variables linked to initiation of tobacco. In order to avoid complexities caused by consideration of younger adults who may only recently have initiated cigarette smoking, possibly only on a temporary basis, attention is limited to adults aged 25 years or more, an age when initiation of cigarettes is less common.

This paper updates an earlier version that described analyses based on follow-up to Wave 3. The current version takes into account comments on the earlier version made by reviewers Chen and Pierce and also includes additional analyses based on follow-up to Wave 4 aimed at providing further understanding ( Chen & Pierce, 2020). There are differences of opinion, described later, in how analyses of smoking cessation should be conducted ( Glasser *et al*., 2020; Pierce *et al*., 2020b) and we consider a variety of approaches.

## Methods

### Original analyses based on data from Waves 1, 2 and 3

Separate sets of analyses have been conducted for three periods, from Wave 1 to Wave 2 (period 1), from Wave 2 to Wave 3 (period 2) and from Wave 1 to Wave 3 (period 3). The analyses are based on individuals with relevant data available at Waves 1, 2 and 3 on smoking and e-cigarette use, and take account of the person-based weights of the baseline population. All analyses are limited to individuals aged 25 years or over at baseline.

Some studies of data from Waves 1 to 3 of the PATH study ( Benmarhnia *et al*., 2018; Pierce *et al*., 2020a; Watkins *et al*., 2020) have limited analyses to quit attempters, but others ( Berry *et al*., 2019; Kalkhoran *et al*., 2020; Verplaetse *et al*., 2019) have not. Although such a limitation more closely mimics randomised control trials ( Pierce *et al*., 2020c) we preferred not to do so in our original analyses, and to avoid bias by adjusting for aspects of quitting in analyses. Our approach uses a larger sample size and provides results that are more representative of the whole population of baseline smokers.

A current cigarette smoker is a “current established cigarette user” defined as “has ever smoked a cigarette, has smoked more than 100 cigarettes in life time, and currently smokes every day or some days”, while a former cigarette smoker is a “former established cigarette user” defined as “has ever smoked a cigarette, has smoked more than 100 cigarettes in life time, and now does not smoke at all”. Those who are neither current nor former cigarette smokers at baseline are not considered in the analyses.

A current e-cigarette user is a “current established e-cigarette user” defined as “has ever used an e-cigarette, has used fairly regularly and uses every day or some days”, while a former e-cigarette user is a “former established e-cigarette user” defined as “has ever used an e-cigarette, has used fairly regularly, and currently does not use at all”. An ever e-cigarette user is either a current or former e-cigarette user. At Wave 2 those who smoked other e-products (such as e-cigars, e-pipes or e-hookahs) are also included, so the definition relates to e-product rather than e-cigarette use.

For each period, the analyses relate ever e-cigarette use at baseline to the probability of being an established former smoker at follow-up (referred to subsequently as either “quitting” or “quitting during follow-up”), with adjustment for predictor variables measured at baseline. The predictor variables have been selected from a pre-defined list of candidates classified into eight groups: demographics (A); general aspects of smoking (B); aspects of smoking specifically related to quitting (C); smoking by family and friends (D); awareness of hazards of smoking (E); health status (F); alcohol and drugs (G); and others (H).

The specific predictor variables are listed in the Results section, with fuller details of their definition given in the *Extended data.* While the variables were chosen as being suggested by the literature as being related to smoking, the *Extended data* also provides information, based on Wave 1, of their association with ever e-cigarette use. As shown there, ever use was highly significantly (p < 0.001) more frequent in the young and in females, and after adjustment for age and sex, was also highly significantly related to a range of the predictor variables considered, being less frequent in Hispanics and Blacks, and more frequent in those with more income or education, those who ever use other tobacco products, those who have a perceived greater need for tobacco, those who have tried to quit more often, those who plan to quit, those who find it hard to stop smoking and those who have used quitting aids. Users were also clearly more likely to have significant problems more recently with sleeping, anxiety and distress, to see a doctor more often, to use the internet often, and to use various different types of drugs (but not cocaine or crack). At most weak relationships were seen with smoking by family and friends, awareness of the hazards of smoking, use of alcohol, body mass index, or self-perception of physical health or quality of life. Little relationship was also seen between ever e-cigarette use and daily cigarette consumption, a finding which was reported earlier ( Lee *et al*., 2020) where it was suggested that it was explained by smokers taking up e-cigarettes having higher consumption initially, reduced by partial replacement of cigarettes by e-cigarettes.

Where the baseline of the period studied is Wave 1, the values of the predictor variables used are as recorded at Wave 1. Where it is Wave 2, the values of some variables are amended to take into account data from Wave 1, as described in the *Extended data*.

For each period, the analysis was conducted in seven stages, preliminary counts and six further steps, each involving weighted logistic regression analyses.

**Counts**      Restricted to individuals who were current cigarette smokers at baseline, a frequency table was prepared, separated by quitting during follow-up, of e-cigarette use at baseline by each of the adjustment variables. Missing values are shown, to indicate variables with high levels of missing values requiring special consideration in analysis.

**Step 1**      This is conducted in eight parts, each part corresponding to a group of predictor variables (A to H). For each part, the regressions first relate each predictor variable individually to quitting, with stepwise forward multiple regressions then carried out, with the most significant predictor variable introduced first, then the next most significant, and so on, until no more variable can be added that is significant at p < 0.01.

**Step 2**      This is in three sections, each involving stepwise forward multiple regressions. The first section considers all the variables found to be significant in Step 1 from groups A, B and C, the second considers those significant from groups D, E and F, and the third those significant from groups G and H.

**Step 3**      A final stepwise regression considers all the predictor variables remaining as significant in step 2. This generates a final list of predictors to be considered when relating ever e-cigarette use to quitting.

                Each analysis in steps 1 to 3 is restricted to those with non-missing data for all the predictor variables considered in the particular analysis.

                The final three steps are then based on all individuals with data on all the predictor variables in the final list.

**Step 4**      An unadjusted analysis relates ever e-cigarette use to quitting.

**Step 5**      Stepwise regression analyses are run, introducing the predictor variables in the final list first, and then adding ever e-cigarettes as a predictor.

**Step 6**      Stepwise regressions similar to those in step 5 are run, but introducing ever e-cigarette use first rather than last.

The principal results produced by the regression analyses are the odds ratios (ORs) and 95% confidence intervals (CIs) relating to each predictor of interest, and the significance of introducing that predictor into the model.

While the main analyses relate quitting cigarettes during follow-up to ever e-cigarette use at baseline and the predictor variables considered include use of nicotine products other than cigarettes or e-cigarettes, four sensitivity analyses (S1 to S4) were also conducted, which are intended to give additional information on how dependent the ORs derived in the main analysis are on exactly how they are conducted. S1 restricts attention to individuals who have never used other nicotine products; S2 links quitting to current (rather than ever) e-cigarette use at baseline; S3 adjusts, where necessary, for variables which take account of data recorded at the end of follow-up rather than just at baseline; and S4, which applies only to the analyses based on quitting between Waves 1 and 3, additionally adjusts for whether the individual had already quit by Wave 2.

In each of S1 to S4 the analyses run were as in steps 4 to 6 of the main analyses and used the final set of predictor variables derived for the period they related to.

For most of the 55 predictor variables considered, there were relatively few missing values, and the regressions could be run excluding the individuals with missing values for the predictors considered without material loss of power. However, for two predictors, where there were about 8% of missing values, individuals with missing data were assigned average values. Thus, for household income in the past 12 months, where data were recorded in five increasing levels, individuals recorded as unknown were assigned an income in the third level, $25,000 to $49,999, while for poverty status, where data were recorded in three levels, <100%, 100–199% and 200+% of the poverty guideline, individuals recorded as unknown were assigned a status in the second level. For living with a regular smoker who smoked inside your home during childhood, where about 16% of individuals were classified as “not ascertained” rather than “yes” or “no”, this answer was included as a separate level, thus the predictor was treated in analysis as having three levels.

For some predictors with multiple levels, the regression analyses were based on a single trend variable. This was only appropriate where the predictor variable represented increasing (or decreasing) levels of a characteristic.

Generally, the analyses were based on the values of predictors as recorded at the baseline Wave. Where the baseline Wave was Wave 2, however, and data were not available at Wave 2, Wave 1 data were used if appropriate. Also, if the Wave 2 predictor related to ever having done something, particularly when the variable concerned action in the last 12 months, individuals were counted as ever having done so if this was reported at Wave 1 or 2.

Further details of the process, particularly for the calculation of numbers of cigarettes per day, are given in the *Extended data*.

### Additional analyses based on data from Waves 1 to 4

While our original analyses only consider overall quitting, our additional analyses separately consider sustained quitting (quitting seen at all follow-up years), temporary quitting (quitting seen at some follow-up years, but not the final one) and delayed quitting (quitting seen at final follow-up, but not all other years since baseline). We also carry out sensitivity analyses with quitting defined as having quit for 30 days at the relevant Wave, and restricting attention to smokers attempting to quit. For comparative purposes, we also carry out analyses comparing rates of sustained, temporary and delayed quitting smoking in smokeless tobacco users and snus users. Much of the methodology is as described for the analyses based on data from Waves 1, 2 and 3.

Separate sets of analyses have been carried out for three periods: from Wave 1 (baseline) to Waves 2 and 3 (follow-up period); from Wave 2 to Waves 3 and 4; and from Wave 1 to Waves 2, 3 and 4. Each analysis is based on individuals with relevant data available on smoking and e-cigarette use at all the Waves considered in that analysis, is limited to individuals aged 25 years or over at baseline, and takes account of the person-based weights of the baseline population. Each analysis restricts attention to baseline established cigarette smokers as defined earlier and compares ever to never regular e-cigarette users at baseline in regard to the probabilities of persistent quitting, temporary quitting or delayed quitting. Adjustment for potential confounding variables is as described above, with logistic regression analyses being carried out to determine which of a list of candidate predictors should be included in the model relating e-cigarette use to the three different definitions of quitting. 

For each follow-up period, four sensitivity analyses have been carried out:

**Sensitivity analysis 5**:  The definition of quitting relates to being a 30-day quitter at the relevant Wave, the analysis otherwise being identical;

**Sensitivity analysis 6**:  The analyses are restricted to those cigarette smokers who ever quit or attempted to quit in the year following baseline;

**Sensitivity analysis 7**:  Instead of comparing never and ever users of e-cigarettes, never and ever users of smokeless tobacco are compared.

**Sensitivity analysis 8**:  Here, never and ever users of snus are compared.

## Results

### Analyses based on data from Waves 1, 2 and 3

Table 1 shows the predictor variables used in the final regression analysis or excluded at various stages of the preliminary analyses.

**Table 1.** Predictor variables included in the final regression analysis (Y) or excluded at steps 1, 2 or 3 (X1, X2, X3).

	Levels of variable ^a^	Wave 1 to 2 quitting	Wave 2 to 3 quitting	Wave 1 to 3 quitting
All smokers at baseline		8,924	7,825	8,924
Not followed at subsequent waves ^b^		2,421 (27.1%)	978 (12.5%)	2,434 (27.3%)
Smokers at baseline		6,503	6,847	6,490
Quit by follow-up		655 (10.1%)	633 (9.2%)	901 (13.9%)
Demographics (A)				
Age range	5	Y	X3	X2
Gender	2	X1	X1	X1
Hispanic origin	2	X2	X2	X2
Race	3	X1	X1	X1
Census region	4	X1	X1	X1
Total household income	5T	Y	Y	Y
Poverty status	3T	X1	-	X1
Total number in the household	5T	X1	-	X1
Highest grade level of school completed	6T	X2	Y	X2
Currently enrolled in a degree program	2	X1	X1	X1
Current employment status	8	X1	X1	X1
Aspects of smoking – general (B)				
Age range started smoking cigarettes fairly regularly	6	X1	X1	X1
Current someday cigarette smokers	2	Y	Y	Y
Cigarettes per day	C	Y	X3	Y
Ever used other tobacco products	2	X1	X1	X1
Frequently crave tobacco product(s)	5T	X1	X1	X1
Usually wants to smoke/use tobacco right after waking	5T	Y	Y	Y
After not smoking for a while, need to smoke to avoid discomfort	5T	X1	X1	X1
Can only go a couple of hours without smoking/tobacco	5T	X1	X1	X1
Aspects of smoking – specifically related to quitting (C)				
Have tried to quit completely	2	Y	Y	Y
Would find it hard to stop smoking/tobacco for a while	5T	X2	X2	X2
Times stopped smoking for one day or more in past year	4T	X1	X1	X1
Ever used a nicotine patch, gum, inhaler, nasal spray, lozenge or pill	2	X1	X1	X1
Ever used Chantix, varenicline or bupropion (Wellbutrin, Zyban)	2	X1	X1	Y
Plans to quit smoking/using tobacco product(s) for good	2	X1	X1	X1
Smoking/using tobacco product(s) really helps me feel better if feeling down	5	X1	X1	X1
Extent disapproval of smoking from friends and family led to thinking about quitting in past year	3T	X1	-	X1
Smoking by family and friends (D)				
Rules about smoking a combustible tobacco	3T	Y	X3	Y
Anyone who lives with you now smoke cigarettes	2	X3	X1	X2
Most people I spend time with are tobacco users	5T	X3	X3	X2
Lived with regular smoker who smoked inside your home during childhood	3	X1	Y	X2
Awareness of hazards of smoking (E)				
How often have you seen a list of chemicals in tobacco products in last 12 months	5T	X1	X1	X1
How often noticed health warnings on cigarette packages in past 30 days	5T	X1	X1	X1
Overall opinion of tobacco	5T	Y	X3	X2
Perception of harmfulness of cigarettes to health	5T	X1	X1	Y
Health status (F)				
Saw a medical doctor in past 12 months	2	X2	X1	X1
Body mass index	C	Y	X3	X1
Self-perception of physical health	5T	X1	X2	X1
Self-perception of quality of life	5T	X3	X3	X3
Last time significant problems with:				
Feeling very trapped, lonely, sad, etc.	4T	X1	X1	X1
Sleep troubles	4T	X1	X1	X1
Feeling very anxious, nervous, tense, etc.	4T	X1	X1	X1
Becoming very distressed with something reminded of past	4T	X1	X1	X1
Alcohol and drugs (G)				
Ever used alcohol	2	X1	X1	X1
Last time used alcohol or other drugs weekly or more often	5T	X1	X1	X1
Days drank an alcoholic beverage in past 30 days	31T	X1	X1	X1
Ever used marijuana, hash, THC or grass	2	X1	X1	X1
Ever used unprescribed Ritalin or Adderall	2	X1	X1	X1
Ever used unprescribed painkillers, sedatives or tranquilizers	2	X1	X1	X1
Ever used cocaine or crack	2	X1	X1	X1
Ever used stimulants like methamphetamine or speed	2	X1	X1	X1
Ever used any other drugs like heroin, inhalants, solvents, hallucinogens	2	X1	X1	X1
Other (H)				
Hours spent watching TV on a typical day	5T	X1	X3	X3
How often uses the Internet	7T	X3	-	X3
Time a day on social media sites	5T	-	X1	-

^a^C = continuous variable, T = treated as a linear trend variable in regressions

^b^These numbers include a few missing values related to e-cigarette status at baseline and smoking status at subsequent waves

For the analyses based on Waves 1 and 2, for example, 54 predictors were considered, 11 in group A, 8 in B, 8 in C, 4 in D, 4 in E, 8 in F, 9 in G, and 2 in H. Of the 55 predictors listed in Table 1, there was one variable in group H with no Wave 1 data. Of the 54 predictors considered in the Wave 1 and 2 analyses, 37 were excluded at step 1, marked X1 in Table 1. A further 4 were excluded at step 2 (X2). This left 13 variables considered in step 3, of which 4 were excluded (X3), with 9 included in the final model (Y).

For the analyses based on Waves 2 and 3 there were data available on 51 predictors, with 45 excluded (34 X1, 3 X2 and 8 X3) and 6 included in the final model. For those based on Waves 1 and 3 there were data on 54 predictors, with 46 excluded (35 X1, 8 X2 and 3 X3) and 8 included in the final model. 

Table 2 summarises the results of the main analyses. Each analysis was based on between 6,000 and 7,000 adults with the percentage quitting varying from 9.1% to 13.1%. The unadjusted gateway-out effect varied from 1.29 to 1.52 in the three analyses. Adjustment only slightly reduced the estimates, the fully adjusted ORs being 1.23 (95%CI 0.94-1.61) for Wave 1 to 2, 1.51 (1.24-1.85) for Wave 2 to 3, and 1.39 (1.11-1.74) for Wave 1 to 3.

**Table 2.** Effect of adjustment on the OR (95% CI) for the relationship of ever regular e-cigarette use ^a^ to quitting.

	Wave 1 to 2 quitting	Wave 2 to 3 quitting	Wave 1 to 3 quitting
Unweighted numbers			
Total – in baseline	6,503	6,847	6,490
Excluded from final regression	262 (4.0%)	89 (1.3%)	175 (2.7%)
Included in final regression	6,241	6,758	6,315
Total ever e-cigarette users – in baseline	727	1,306	726
Excluded from final regression	24 (3.3%)	12 (0.9%)	16 (2.2%)
Included in final regression	703	1,294	710
Quit	78 (11.1%)	160 (12.4%)	118 (16.6%)
Total never e-cigarette users – in baseline	5,776	5,541	5,764
Excluded from final regression	238 (4.1%)	77 (1.4%)	159 (2.8%)
Included in final regression	5,538	5,464	5,605
Quit	503 (9.1%)	456 (8.3%)	711 (12.7%)
Weighted ORs and 96% CIs			
Unadjusted	1.29 (1.01-1.66)	1.52 (1.26-1.83)	1.47 (1.19-1.82)
Adjusted for four most important variables	1.17 (0.90-1.52)	1.54 (1.26-1.88)	1.43 (1.14-.1.78)
Adjusted for all variables included in final list	1.23 (0.94-1.61) [9 variables]	1.51 (1.24-1.85) [6 variables]	1.39 (1.11-1.74) [8 variables]

^a^Where the baseline is Wave 1, the predictor is ever regular e-cigarette use, where it is Wave 2, it is ever regular e-product use

Table 3 shows the full models used, showing the effect estimates for each of the predictor variables used to adjust the relationship of ever regular e-cigarette use to quitting. Where the same adjustment variable was included in each model, the effect estimates were generally quite similar and always in the same direction. As regards aspects of smoking, smokers were found to be less likely to quit if they were everyday smokers, were more likely to smoke right after waking up, had not previously tried to quit, smoked more cigarettes per day, lived in a home with more relaxed rules about smoking, lived with a smoker in childhood, had a better opinion of tobacco, or had a lesser perception of cigarettes as harmful. They were also less likely to quit if they had ever used the pharmaceutical aids to quitting Chantix, varenicline or buproprion (Wellbutrin, Zyban). Smokers were also less likely to quit if they were worse off and worse educated. Older age (particularly above age 74 years) and greater BMI were also associated with a greater likelihood to quit.

**Table 3.** ORs related to quitting cigarettes in the final models used in the main analysis.

	Wave 1 to 2 quitting	Wave 2 to 3 quitting	Wave 1 to 3 quitting
Ever regularly used e-cigarette = yes	1.23 (0.94-1.61)	1.51 (1.24-1.85)	1.39 (1.11-1.74)
Age range			
25–34	Reference	Not included	Not included
35–44	0.95 (0.74-1.21)		
45–54	0.85 (0.66-1.10)		
55–64	1.20 (0.92-1.57)		
65–74	1.42 (0.98-2.06)		
75+	3.31 (1.84-5.97)		
Total household income (per level increasing)	1.14 (1.05-1.23)	1.16 (1.07-1.25)	1.21 (1.13-1.29)
Current some day smoker = no	0.43 (0.34-0.54)	0.36 (0.30-0.44)	0.52 (0.43-0.64)
Highest grade or level at school completed (per level increasing)	Not included	1.10 (1.03-1.18)	Not included
Per cigarette per day	0.97 (0.96-0.99)	Not included	0.97 (0.96-0.98)
Usually wants to smoke/use tobacco right after waking up (per level increasing)	0.89 (0.83-0.95)	0.79 (0.74-0.84)	0.85 (0.81-0.90)
Have tried to quit completely = no	0.62 (0.51-0.75)	0.64 (0.53-0.77)	0.69 (0.58-0.82)
Ever used Chantix, varenicline or buproprion (Wellbutrin, Zyban)	Not included	Not included	0.78 (0.65-0.94)
Rules about smoking combustible tobacco at home (per level of decreasing stringency)	0.77 (0.68-0.88)	Not included	0.78 (0.70-0.87)
Lived with a regular smoker who smoked inside your home in childhood = no	Not included	1.41 (1.18-1.68)	Not included
Overall opinion of tobacco (per level of increasing negativity)	1.17 (1.07-1.29)	Not included	Not included
Perception of harmfulness of cigarettes to health (per level increasing)	Not included	Not included	1.13 (1.03-1.24)
Per unit of body mass index	1.02 (1.01-1.03)	Not included	Not included

Table 4 summarises the results of the sensitivity analyses, showing the estimated ORs in each case from the fully adjusted analysis. The first line of results (“Main model”) repeats the estimates shown in Table 3.

**Table 4.** Comparing adjusted ORs for the effect of e-cigarette use on quitting in the main and sensitivity analyses.

	Wave 1 to 2 quitting		Wave 2 to 3 quitting		Wave 1 to 3 quitting	
	N(n)	OR (95% CI)	N(n)	OR (95% CI)	N(n)	OR (95% CI)
Main model	581 (78)	1.23 (0.94-1.61)	616 (160)	1.51 (1.24-1.85)	829 (118)	1.39 (1.11-1.74)
Sensitivity analysis 1 (Omitting ever users of other nicotine products)	148 (16)	2.04 (1.15-3.62)	122 (20)	1.69 (0.96-2.99)	217 (25)	2.22 (1.38-3.57)
Sensitivity analysis 2 (Linking quitting to current rather than ever e-cigarette use at baseline)	581 (64)	1.41 (1.06-1.89)	615 (89)	1.30 (1.01-1.67)	829 (95)	1.56 (1.21-2.00)
Sensitivity analysis 3 (Adjusting for variables taking account of data recorded at both baseline and end of follow-up)	655 (82)	1.20 (0.92-1.57)	591 (160)	1.66 (1.35-2.04)	901 (121)	1.43 (1.14-1.79)
Sensitivity analysis 4 (Adjust also for quitting by Wave 2)		Not applicable		Not applicable	443 (64)	1.43 (1.11-1.84)

N = total number quitting n = number of quitters among e-cigarette users (current users in sensitivity analysis 2, ever users otherwise)

Sensitivity analysis 1 excludes those who had ever used other nicotine products. The number of quitters is substantially reduced, as is the number using e-cigarettes (or e-products). However, the OR is increased, with ever regular users of e-cigarettes about twice as likely to quit cigarettes by the end of the follow-up period, though the CIs of the ORs are relatively wide.

In sensitivity analysis 2, quitting is linked to current rather than ever e-cigarette use. Here the ORs tend to be somewhat higher than in the main analysis (though not for the Wave 2 to 3 analysis).

The results for both sensitivity analysis 1 and 2 seem consistent with smokers being more likely to quit if, at baseline, e-cigarettes formed a more important part of the total tobacco use.

Sensitivity analysis 3 adjusts, where necessary, for variables which are modified to take account of data recorded at the end of follow-up, and not just at baseline, in an attempt to minimise “residual confounding”. The ORs were quite similar to those in the main analysis for Wave 1 to 2, or Wave 1 to 3 quitting, but were somewhat increased for Wave 2 to 3 quitting.

Sensitivity analysis 4, only applicable to the Wave 1 to 3 quitting analyses, adjusted also for having quit by Wave 2. This slightly increased the estimate from the main analysis.

### Analyses based on data from Waves 1 to 4

Table 5 shows which predictor variables were included in the regression analysis for the three main analyses and all the sensitivity analyses. Seven predictor variables were included in at least 10 of the 15 regression analyses, with a further three included in at least five, and 11 other predictors occurring in at least one. The direction of the associations was generally consistent, with quitting cigarettes more likely in those who, for example, smoked someday rather than every day, had previously tried to quit, who were less likely to smoke right after waking up, were aged 75 or more, had a higher income, and who smoked less cigarettes a day.

**Table 5.** Predictor variables included in the regression analyses in the order they were included.

Variable	Levels of Variable	Wave 1 to 2/3 quitting					Wave 2 to 3/4 quitting					Wave 2/3/4 quitting				
		M	S5	S6	S7	S8	M	S5	S6	S7	S8	M	S5	S6	S7	S8
Age range	6	8		6	8	6	7		6	7	7	8		8	8	8
Gender	2							9								
Total household income	5T	7	3	4	10	4	1	1	1	1	1	10	4	5	10	10
Currently enrolled in a degree program	2							6								
Current someday cigarette smokers	2	1	1	1	1	1	2	2	2	2	2	1	1	1	1	1
Cigarettes per day	C	2	2		2							2	2	6	2	2
Frequently crave tobacco product(s)	5T			2		2								2		
Usually wants to smoke/use tobacco right after waking	5T	5			7		3	4	3	3	3	7			7	7
Have tried to quit completely	2	3			4		5	5		5	4	3	8		4	3
Would find it hard to stop smoking/tobacco for a while	5T		6										6			
Plans to quit smoking/using tobacco product(s) for good	2			3		3								3		
Rules about smoking a combustible tobacco	3T	4	7	5	3	5						4	7	4	3	4
Most people I spend time with are tobacco users	5T				5							5			5	5
Lived with regular smoker who smoked inside your home during childhood	3						4	3	4	4	5					
How often have you seen a list of chemicals in tobacco products in last 12 months	5T								7							
How often noticed health warnings on cigarette packages in past 30 days	5T													9		
Overall opinion of tobacco	5T	6	5		6							6	5		6	6
Body mass index	C	9		7	9	7	6	8	5	6	6	9		7	9	9
Becoming very distressed with something reminded of past	4T							7								
Ever used marijuana, hash, THC or grass	2		4										3			
Hours spent watching TV on a typical day	5T						8				8					
Total adjustment variables		9	7	7	10	7	8	9	7	7	8	10	8	9	10	10

C = continuous variable, M = Main analysis, S = Sensitivity analysis, T = treated as a linear trend in regressions

Table 6 shows the results of the main analyses. The nine unadjusted ORs for each combination of period and type of quitting were all greater than 1.00, ranging from 1.10 for temporary quitting for Wave 1 to 2/3, to 1.68, for temporary quitting for Wave 2 to 3/4. Adjustment for all the variables included in the final list generally slightly decreased the ORs, though there were some exceptions, with the adjusted OR significant (at p < 0.05) in seven of the nine cases. Notably all the fully adjusted ORs for sustained quitting were positive and significant, with estimates close to 1.50 for each period studied.

**Table 6.** Effect of adjustment on the OR (95% CI) for the relationship of ever regular e-cigarette use ^a^ to quitting.

Type of quitting	Statistic	Wave 1 to 2/3 quitting	Wave 2 to 3/4 quitting	Wave 1 to 2/3/4 quitting
Delayed	Number in baseline in final regression	5,660	5,346	4,956
	Number quitting	448	392	606
	Unadjusted	1.50 (1.13-1.97)	1.28 (1.00-1.64)	1.52 (1.19-1.94)
	Adjusted for four most important variables	1.36 (1.02-1.82)	1.29 (1.00-1.66)	1.41 (1.10-1.82)
	Adjusted for all variables included in final list	1.38 (1.03-1.85)	1.25 (0.97-1.62)	1.43 (1.10-1.85)
Temporary	Number in baseline in final regression	5,413	5,149	4,699
	Number quitting	201	195	349
	Unadjusted	1.10 (0.71-1.70)	1.68 (1.22-2.32)	1.34 (0.97-1.86)
	Adjusted for four most important variables	0.91 (0.58-1.42)	1.74 (1.25-2.43)	1.20 (0.86-1.69)
	Adjusted for all variables included in final list	0.93 (0.59-1.46)	1.60 (1.14-2.25)	1.26 (0.89-1.77)
Sustained	Number in baseline in final regression	5,602	5,298	4,622
	Number quitting	380	344	272
	Unadjusted	1.48 (1.10-1.99)	1.49 (1.16-1.91)	1.29 (0.89-1.86)
	Adjusted for four most important variables	1.41 (1.03-1.93)	1.59 (1.22-2.06)	1.33 (0.91-1.96)
	Adjusted for all variables included in final list	1.51 (1.09-2.08)	1.48 (1.13-1.94)	1.52 (1.02-2.26)

^a^Where the baseline is Wave 1, the predictor is ever regular e-cigarette use, where the baseline is Wave 2, the predictor is ever regular e-product use

Table 7 summarises the adjusted results for the main and sensitivity analyses, again for each combination of period and type of quitting. Of the 45 ORs, 42 are positive (> 1.0) with 20 of these statistically significant, and three are negative (< 1.0) with none of these significant. Associations are clearly evident for 30-day quitting, and when restricted by those attempting to quit in the year following baseline. Interestingly, associations are seen, in some cases larger than for e-cigarette use, both for smokeless tobacco and for snus use.

**Table 7.** Comparing adjusted ORs for the effect of e-cigarette, smokeless tobacco or snus use on quitting in the main and sensitivity analyses.

		Wave 1 to 2/3 quitting		Wave 2 to 3/4 quitting		Wave 1 to 2/3/4 quitting	
Type of quitting	Model	N (n)	OR (95% CI)	N (n)	OR (95% CI)	N (n)	OR (95% CI)
Delayed	Main	448 (65)	1.38 (1.03-1.85)	392 (91)	1.25 (0.97-1.62)	606 (89)	1.43 (1.10-1.85)
	Sensitivity 5	377 (56)	1.50 (1.11-2.04)	352 (84)	1.28 (0.99-1.67)	538 (83)	1.53 (1.18-1.99)
	6	261 (40)	1.26 (0.86-1.86)	222 (61)	1.21 (0.87-1.66)	344 (50)	1.21 (0.85-1.72)
	7	449 (41)	1.16 (0.71-1.90)	396 (42)	1.07 (0.77-1.51)	603 (61)	1.42 (1.05-1.91)
	8	476 (21)	1.79 (0.91-3.53)	394 (18)	1.16 (0.68-1.97)	608 (22)	1.32 (0.80-2.19)
Temporary	Main	201 (24)	0.93 (0.59-1.46)	195 (55)	1.60 (1.14-2.25)	349 (48)	1.26 (0.89-1.77)
	Sensitivity 5	142 (20)	1.29 (0.79-2.09)	134 (39)	1.69 (1.14-2.53)	258 (37)	1.31 (0.90-1.91)
	6	210 (24)	0.93 (0.58-1.47)	195 (55)	1.24 (0.87-1.75)	282 (44)	1.48 (1.01-2.16)
	7	199 (18)	1.16 (0.69-1.96)	192 (30)	1.79 (1.19-2.68)	345 (32)	1.14 (0.75-1.73)
	8	208 (9)	1.96 (0.97-3.96)	193 (19)	3.29 (1.98-5.46)	347 (16)	1.93 (1.08-3.43)
Sustained	Main	380 (54)	1.51 (1.09-2.08)	344 (86)	1.48 (1.13-1.94)	272 (33)	1.52 (1.02-2.26)
	Sensitivity 5	289 (35)	1.36 (0.94-1.97)	243 (62)	1.50 (1.11-2.02)	206 (22)	1.46 (0.93-2.31)
	6	425 (57)	1.45 (1.03-2.05)	350 (87)	1.17 (0.88-1.56)	281 (33)	1.45 (0.95-2.22)
	7	385 (38)	1.57 (1.04-2.35)	343 (37)	1.04 (0.71-1.50)	269 (28)	1.76 (1.14-2.72)
	8	423 (19)	2.30 (1.28-4.14)	345 (12)	0.80 (0.43-1.52)	272 (11)	1.99 (0.99-3.98)

N = total number quitting for the given type n = number of quitters among users Main Comparison of ever and never regular e-cigarette users Sensitivity 5 As main, but relates to 30-day quitting at the relevant wave Sensitivity 6 As main, but excluding those not attempting to quit in first year of follow-up Sensitivity 7 As main, but comparison of ever and never smokeless tobacco users Sensitivity 8 As main, but comparison of ever and never snus users

## Discussion

### Discussion of results based on Waves 1, 2 and 3

The first set of analyses described in this report summarise evidence from Waves 1, 2 and 3 of the US PATH study relating to the possibility that e-cigarette use may increase the likelihood of smokers quitting cigarettes. All of the adjusted ORs estimated, which as shown in Table 4 varied between 1.20 and 2.22, were consistent with this possibility, although not all the estimates were statistically significant at p < 0.05. Compared to the estimates from the main model, which related ever e-cigarette use at baseline to quitting by follow-up, ORs were increased (though based on far fewer quitters) when those who had ever used other products were omitted from the analysis. The ORs were also increased, in the analysis with Wave 1 as the baseline, when quitting was linked to current rather than ever e-cigarette use. In both the sensitivity analyses where the ORs were increased, e-cigarette use would have formed a greater proportion of current tobacco use at baseline.

Eight other related analyses based on the first three Waves 1 of the PATH study have previously been published. The first seven analyses summarised below ( Benmarhnia *et al*., 2018; Berry *et al*., 2019; Glasser *et al*., 2021; Kalkhoran *et al*., 2020; Kurti *et al*., 2020; Verplaetse *et al*., 2019; Watkins *et al*., 2020) are consistent with e-cigarette use increasing the probability of quitting cigarettes, despite variation in whether Wave 3 data has been used or not, whether analyses are restricted to those attempting quitting at baseline, the definition of abstinence used, the confounding variables adjusted for, the age range of the population studied, and other analytical details. However, a final analysis by Pierce *et al*. (2020a) only reported a small and non-significant increase in quitting related to e-cigarette use. 

An analysis of 3,093 quit attempters based on adult data from Waves 1 and 2 ( Benmarhnia *et al*., 2018) considered two endpoints – abstinence from smoking for at least 30 days and reduced cigarette consumption – and reported a significant increase in both endpoints related to using e-cigarettes to quit during the previous year, but no significant increase in either endpoint related to the use of approved pharmaceutical aids.

Another analysis based on Waves 1 and 2 ( Berry *et al*., 2019), here limiting attention to adults aged 25 years or more, studied factors related to 30-day cigarette cessation and to at least a 50% reduction in cigarette consumption in multivariable logistic regression analyses, which included a number of the variables included as predictors in our analyses. While the model included e-cigarette use, this was defined not at baseline, but as new e-cigarette use at Wave 2. In this analysis large ORs were reported for everyday e-cigarette use both for cessation (7.88, 95% CI 4.45-13.95) and for a 50% reduction in cigarette consumption (5.70, 3.47-9.35).

A further analysis based on Waves 1 and 2 ( Verplaetse *et al*., 2019) considered adults aged 18+ years and reported that, compared to those who had never used e-cigarettes at Wave 1, quitting was increased in Wave 1 daily users (OR 1.56. 95%CI 1.12-2.18) but not in Wave 1 nondaily users (0.83, 0.68-1.02). Age, race and education were the only adjustment variables considered.

Based mainly on data from Waves 1 and 2, an analysis limited to women aged 18-49 years ( Kurti *et al*., 2020) and adjusted for demographic and psychosocial characteristics and pregnancy status concluded that use of e-cigarettes by smokers at Wave 1 was associated with an increased odds of quitting at Wave 2.

Analyses based on data from Waves 1, 2 and 3 ( Watkins *et al*., 2020), conducted separately for adults aged 18-24 years and 25+ years, studied the relation of a variety of cessation strategies to short-term cessation (quit at Wave 2) and long-term cessation (quit at both Waves 2 and 3). Adjustments were made for a range of covariates. The authors reported that “substitution with e-cigarettes” did not predict long-term cessation but predicted short-term cessation for older daily smokers of 5 or more cigarettes a day.

An analysis based on data for adults from Waves 1, 2 and 3 ( Kalkhoran *et al*., 2020), related current e-cigarette use at Wave 1 (defined as daily, non-daily or none) in cigarette smokers at Wave 1 to three cigarette abstinence endpoints: at Wave 2, at Wave 3 or at Waves 2 and 3 (prolonged abstinence). Adjustments were made for a fixed set of variables: age, sex, race/ethnicity, education, income, cigarettes per day, and having a first cigarette within 30 minutes of waking. Non-daily e-cigarette use was only associated with a small, non-significant increase in each of the abstinence endpoints, but daily e-cigarette use was associated with a clear increase in all three endpoints, with adjusted ORs of 1.53 (95%CI 1.04-2.23) for Wave 2 abstinence, 1.57 (1.12-2.21) for Wave 3 abstinence, and 1.77 (1.08-2.89) for abstinence at both Waves 2 and 3. These results particularly seem quite similar to ours.

Another analysis of data from Waves 1, 2 and 3 ( Glasser *et al*., 2021) found that smokers using e-cigarettes daily or increasing to daily use over the 3 waves were 2–4 times more likely to have quit smoking, both in the short and the long-term (p < 0.001). However, smokers using e-cigarettes less often or not at all were less likely to quit.

A fourth analysis based on data from Waves 1, 2 and 3 ( Pierce *et al*., 2020a) restricted attention to adult (ages 18+) smokers identified at Wave 1 who reported a quit attempt before Wave 2 and completed Wave 3. 12-month abstinence at Wave 3 among e-cigarette users was slightly but non-significantly reduced as compared both to users of pharmacotherapy to quit or no product.

The strengths of our work include the use of a prospective study design based on a study population which is representative of the US, and analyses which take account of a very large number of other predictors of quitting, and restrict attention to established e-cigarette use.

Limitations relate to the relatively small number of quitters, leading to the decision not to study heterogeneity of the results by basic variables, such as sex, race or age group. Our decision to limit attention to those aged at least 25 years was based on the desire not to include young smokers whose smoking habits were not well established. 

### Discussion of additional results based on Waves 1 to 4

A publication based on the PATH study that reported a positive association of e-cigarette use with quitting by Glasser *et al*. (2021) was criticised by Pierce *et al*. (2020b), who themselves had found no significant association ( Pierce *et al*., 2020a), on the grounds that they included smokers not wanting to quit or making a quit attempt, and used a design that did not assess e-cigarette exposure before the smoking cessation outcome was assessed. In their reply, Glasser *et al*. (2020), pointed out that asking different questions requires different methods, and that while the study by Pierce *et al*. (2020a) was “framed as an intervention study”, their study “attempted to answer a broader question; the impact of e-cigarettes on cigarette smoking cessation among the full sample of smokers in the PATH study.” Our first set of analyses did assess e-cigarette exposure before the smoking cessation outcome, so no revised analysis was necessary here. However, though our analyses were also an attempt to answer the broader question of Glasser *et al*., we did include a sensitivity analysis (6) in our analyses based on Waves 1 to 4 to show the effect of limiting attention to those considering quitting smoking. Conducting such a sensitivity analysis had also been noted in version 1 of this paper as an option for consideration in further analysis, using the additional data from Wave 4. Version 1 had also noted the possibility of studying sustained quitting, considered in the additional results along with delayed and temporary quitting, and of analysing 30 day quitting, considered in sensitivity analysis 5. 

The additional results based on Waves 1 to 4, illustrate that there is an association of e-cigarette use at baseline with subsequent quitting, whether this be delayed, temporary or sustained, that the association is little affected by considering 30-day quitting rather than not being still an established smoker at the time of interview, and that though the ORs are perhaps reduced slightly by restricting attention to those attempting to quit, they generally remain positive and in some analyses statistically significant.

The additional results show that cigarette smokers who also use smokeless tobacco or also use snus are also more likely to quit smoking, with some of the ORs for snus use larger than those for e-cigarettes. These results seem consistent with the general proposition that, even after detailed adjustment for potential confounding variables, those who both smoke and use a reduced risk tobacco product are more likely to quit smoking than those who do not also use the reduced risk product. 

While it is clear that even more analyses could be run using the PATH study, the conclusions will inevitably be limited by sample size considerations, and a larger and well-designed study could provide a clearer picture.

Two other analyses of e-cigarettes and quitting have been conducted based on Waves 1 to 4 of the PATH study.

In an analysis considering a variety of transitions in tobacco use between Waves, Brouwer *et al*. (2020) reported that cigarette smokers using e-cigarettes were more likely to quit cigarettes than were exclusive cigarette smokers (hazard ratio 1.9, 95%CI 1.6 to 2.3).

In contrast, Chen *et al*. (2020) concluded that “e-cigarettes may not be an effective cessation aid for adult smokers and instead may contribute to nicotine dependence”. They found that among smokers using e-cigarettes to help quit, rates of long-term abstinence were only 2% higher (95%CI −3% to 7%) than in matched smokers not using e-cigarettes, and also that fewer e-cigarette users were long-term abstinent from all nicotine products (Difference −4%, 95%CI −7% to −1%), though this second finding is not specifically related to quitting cigarette smoking.

## Conclusion

Our results clearly suggest that among adults aged 25 years or more, most of whom would not have initiated smoking recently, e-cigarettes may assist in helping smokers to quit, particularly if, at baseline, e-cigarettes form an important part of total tobacco use – i.e. for individuals who at baseline did not use products other than cigarettes or e-cigarettes, and who were current rather than ever e-cigarette users. These conclusions apply whether delayed, temporary or sustained quitting is considered. They are independent of the definition of quitting used, whether all cigarette smokers are considered or only those considering quitting. The results seem consistent with most of the evidence from clinical trials, other analyses of the PATH study, and other epidemiological studies.

## Data availability

### Underlying data

National Addiction & HIV Data Archive Program: Population Assessment of Tobacco and Health (PATH) Study [United States] Public-Use Files (ICPSR 36498). https://doi.org/10.3886/ICPSR36498.v8 ( United States Department of Health and Human Services (USDHHS), 2018).

The data are available under the Terms of Use as set out by ICPSR, which can be accessed when users start the process of downloading the data.

### Extended data

Open Science Framework: Investigating the effect of e-cigarette use on quitting smoking in adults aged 25 or more using the PATH study https://doi.org/10.17605/OSF.IO/5XWQP ( Lee *et al*., 2020).

This project contains the following extended data file:

- Additional file_fuller details regarding the predictor variables used_v2.docx

Data are available under the terms of the Creative Commons Zero “No rights reserved” data waiver (CC0 1.0 Public domain dedication).
