# Supplementary material for: Investigating the effect of e-cigarette use on quitting smoking in adults aged 25 years or more using the PATH study
Source: F1000Res. 2022 Jun 20;9:1099. Originally published 2020 Sep 7. [Version 3] doi: 10.12688/f1000research.26167.3 (PMC9214270; doi:10.12688/f1000research.26167.3)
Supplement: Peer review history

# Peer review: Reviewer response for version 3

**DOI:** 10.5256/f1000research.135096.r141345

**Published:** 2022-07-06

**Authors:** John P. Pierce, Tarik Benmarhnia, Karen Messer

**Affiliations:** 1 Moores Cancer Center, Herbert Wertheim School of Public Health and Human Longevity Science, University of California, San Diego, San Diego, CA, USA; 2 Division of Epidemiology. Herbert Wertheim School of Public Health and Human Longevity Science, University of California San Diego, San Diego, CA, USA; 3 Scripps Institution of Oceanography, University of California San Diego, San Diego, CA, USA; 4 Herbert Wertheim School of Public Health and Moores Cancer Center, University of California, San Diego, San Diego, CA, USA

The authors disagree with all of our points without refutation and have announced their intention not to revise in response to our concerns. This includes our concerns on two critical scientific methods issues: 1) by not restricting the study population to those who had made a quit attempt, the study is tainted by reverse causation and 2) that the variable selection strategy employed opens the study to major collider bias. Our prior evaluation stands. This paper cannot be approved.

Is the work clearly and accurately presented and does it cite the current literature?

Partly

If applicable, is the statistical analysis and its interpretation appropriate?

Partly

Are all the source data underlying the results available to ensure full reproducibility?

Yes

Is the study design appropriate and is the work technically sound?

No

Are the conclusions drawn adequately supported by the results?

No

Are sufficient details of methods and analysis provided to allow replication by others?

Yes

Reviewer Expertise:

1. John Pierce, behavioral epidemiology with an emphasis on tobacco use 2. Tarik Benmarhnia, epidemiological methods 3. Karen Messer, statistical methods

We confirm that we have read this submission and believe that we have an appropriate level of expertise to state that we do not consider it to be of an acceptable scientific standard, for reasons outlined above.

# Peer review: Reviewer response for version 3

**DOI:** 10.5256/f1000research.135096.r141344

**Published:** 2022-06-30

**Authors:** Riccardo Polosa

**Affiliations:** 1 Center of Excellence for the Acceleration of HArm Reduction (CoEHAR), Department of Clinical and Experimental Medicine, University of Catania, Catania, Italy

I have reviewed this new version. Revisions are appropriate and I have no additional comments.

Is the work clearly and accurately presented and does it cite the current literature?

Yes

If applicable, is the statistical analysis and its interpretation appropriate?

I cannot comment. A qualified statistician is required.

Are all the source data underlying the results available to ensure full reproducibility?

Yes

Is the study design appropriate and is the work technically sound?

Yes

Are the conclusions drawn adequately supported by the results?

Yes

Are sufficient details of methods and analysis provided to allow replication by others?

Yes

Reviewer Expertise:

NA

I confirm that I have read this submission and believe that I have an appropriate level of expertise to confirm that it is of an acceptable scientific standard.

# Peer review: Reviewer response for version 3

**DOI:** 10.5256/f1000research.135096.r141346

**Published:** 2022-06-22

**Authors:** Arielle S. Selya

**Affiliations:** 1 PinneyAssociates, Inc., Pittsburgh, PA, USA

No further comments.

Is the work clearly and accurately presented and does it cite the current literature?

Yes

If applicable, is the statistical analysis and its interpretation appropriate?

Yes

Are all the source data underlying the results available to ensure full reproducibility?

Yes

Is the study design appropriate and is the work technically sound?

Yes

Are the conclusions drawn adequately supported by the results?

Yes

Are sufficient details of methods and analysis provided to allow replication by others?

Yes

Reviewer Expertise:

Tobacco use behavior

I confirm that I have read this submission and believe that I have an appropriate level of expertise to confirm that it is of an acceptable scientific standard.

# Peer review: Reviewer response for version 3

**DOI:** 10.5256/f1000research.135096.r141343

**Published:** 2022-06-21

**Authors:** Alan G. Kaplan

**Affiliations:** 1 Family Physician Airways Group of Canada, University of Toronto, Toronto, ON, Canada

The authors did respond to my responses, other than making it more clinical which they recognized was not in their domain.

Is the work clearly and accurately presented and does it cite the current literature?

Yes

If applicable, is the statistical analysis and its interpretation appropriate?

I cannot comment. A qualified statistician is required.

Are all the source data underlying the results available to ensure full reproducibility?

Yes

Is the study design appropriate and is the work technically sound?

Yes

Are the conclusions drawn adequately supported by the results?

Yes

Are sufficient details of methods and analysis provided to allow replication by others?

Partly

Reviewer Expertise:

NA

I confirm that I have read this submission and believe that I have an appropriate level of expertise to confirm that it is of an acceptable scientific standard.

# Peer review: Reviewer response for version 2

**DOI:** 10.5256/f1000research.54961.r134820

**Published:** 2022-05-20

**Authors:** Arielle S. Selya

**Affiliations:** 1 PinneyAssociates, Inc., Pittsburgh, PA, USA

This manuscript examines the association between e-cigarette use and subsequently discontinuing smoking, using PATH data from adults 25+. E-cigarette use is associated with discontinuing smoking after adjusting for a wide range of covariates, and this association is robust across an impressive range of sensitivity analyses (e.g. omitting users of other tobacco products; using several different outcome measures, including sustained, delayed, and temporary discontinuing smoking; restricting analyses to those who made a quit attempt). While (as the authors note) there have been several similar studies on this question using PATH, a particular advantage of this one in my judgment is including those smokers who did not make a quit attempt (thus capturing some of the accidental switching phenomenon that seems to make e-cigarettes so effective). Another advantage is including former smokers in the analysis, as that captures more people who already successfully switched between waves, as opposed to other studies which subset to who’s still smoking at the time of data collection.

 Major comments:

- Consider changing “quitting” to “discontinuation of smoking” throughout. The word “quitting” has connotations of formal smoking cessation, which involves explicitly setting quit goals, taking certain actions for the purpose of quitting, etc. However, as the authors point out, this does not apply to the entire sample: most smokers at any given time are not immediately planning to quit, if ever. For this reason, the PATH team has used the term “discontinuation of smoking” to make this distinction (see Kasza *et al.*, 2021 ^1^). Using this terminology rather than “quitting” may also help to clarify some of the criticisms to the V1 of this manuscript and the Glasser study (i.e. whether those who made no attempt to quit should be included).
- Is it the case that successful switchers between waves were included in the analysis? I believe this is the case based on including both former and current smokers. If so, that is a great strength of this paper compared to most others (which usually focus only on those who remain current smokers, and thus exclude those who successfully switched to e-cigarettes between waves), and is worth explicitly pointing out.

Minor comments:

- Missing word (?) in Abstract, 1 ^st^ sentence of Results (“ORs of quitting smoking forever e-cigarette use were…”).
- The tables which present ORs could present significant ones (not crossing 1) as boldface for easier presentation of the many results.

Is the work clearly and accurately presented and does it cite the current literature?

Yes

If applicable, is the statistical analysis and its interpretation appropriate?

Yes

Are all the source data underlying the results available to ensure full reproducibility?

Yes

Is the study design appropriate and is the work technically sound?

Yes

Are the conclusions drawn adequately supported by the results?

Yes

Are sufficient details of methods and analysis provided to allow replication by others?

Yes

Reviewer Expertise:

Tobacco use behavior

I confirm that I have read this submission and believe that I have an appropriate level of expertise to confirm that it is of an acceptable scientific standard, however I have significant reservations, as outlined above.

## References

[1] : Association of e-Cigarette Use With Discontinuation of Cigarette Smoking Among Adult Smokers Who Were Initially Never Planning to Quit. *JAMA Network Open* .2021;4(12) : 10.1001/jamanetworkopen.2021.40880 10.1001/jamanetworkopen.2021.40880 PMC871534034962556

# Response

**Published:** 2022-06-10

**Authors:** Peter Lee

**Affiliations:** P.N.Lee Statistics and Computing Ltd, UK

We thank Dr Selya for her comments.

 As regards the first major comment of Dr. Selya, we have not changed “quitting” to “discontinuation of smoking” throughout, as we have already published a very large number of papers using quitting in the sense that we do, and one that is widely understood by epidemiologists. However we have added an extra paragraph (2) in the discussion which explains our terminology. Thus the paragraph starts:

**Note that the term “quitters”, as used here, relates to those who were smoking at baseline, but not at the end of follow-up, even though they may later relapse to smoking. Some prefer the term “discontinuation of cigarette smoking”,**

 the paragraph then going on to cite the reference to Kasza et al., 2021 that Dr. Selya mentioned.

 The same paragraph answers the second major comment of Dr. Selya regarding the inclusion of switchers in the analysis. Thus, the paragraph goes on to state:

**Here, we use “quitting” to relate to cigarette smoking, regardless of use of, or switching to, e-cigarettes or other nicotine products,**

 the paragraph then going on to point out that we

**use “smoking”, not further defined, to relate to cigarettes.**

 Finally the minor comments have been dealt with by:

 1) Correcting the typo of **forever** to **for ever** and;

 2) Making significant ORs in boldface in Tables 2, 3, 4, 6 and 7 with a note in the text about this when introducing Table 2.

 We hope that our paper can now be approved.

# Peer review: Reviewer response for version 2

**DOI:** 10.5256/f1000research.54961.r119744

**Published:** 2022-01-14

**Authors:** Alan G. Kaplan

**Affiliations:** 1 Family Physician Airways Group of Canada, University of Toronto, Toronto, ON, Canada

This study was to review from another larger trial regarding factors to affect smoking cessation that looked at e-cigarette efficacy in such. 

 I found this to be a very difficult study to read, very statistical, which is fine, but not very clinical. You discuss multiple waves, but I am not sure how relevant these are with regards to e-cigarettes and cessation.

 The findings are very important to reinforce the potential value of e-cigarettes for smoking cessation, but there is no balance regarding the potential adverse effects of e-cigarettes which are a concern for many physicians.

 While multiple paramaters are reviewed as possible confounders, other pharmacotherapeutic smoking cessation options are not included.

Is the work clearly and accurately presented and does it cite the current literature?

Yes

If applicable, is the statistical analysis and its interpretation appropriate?

I cannot comment. A qualified statistician is required.

Are all the source data underlying the results available to ensure full reproducibility?

Yes

Is the study design appropriate and is the work technically sound?

Yes

Are the conclusions drawn adequately supported by the results?

Yes

Are sufficient details of methods and analysis provided to allow replication by others?

Partly

Reviewer Expertise:

Respiratory illness and chronic pain

I confirm that I have read this submission and believe that I have an appropriate level of expertise to confirm that it is of an acceptable scientific standard, however I have significant reservations, as outlined above.

# Response

**Published:** 2022-06-10

**Authors:** Peter Lee

**Affiliations:** P.N.Lee Statistics and Computing Ltd, UK

We thank Dr. Kaplan for his comments.

 As regards the comment “I found it to be a very difficult study to read, very statistical, which is fine, but not very clinical” all we can say is that neither of the authors are medically qualified, and both are statisticians, and as there were a large number of analyses to describe, the paper was inevitably rather statistical.

 Dr. Kaplan also says “You discuss multiple waves, but I am not sure how relevant these are with regards to e-cigarettes and cessation.”  Clearly, when studying cessation one needs data at two time points (waves), to have individuals changing from being smokers to non-smokers, i.e. quitting. Our original analyses carried out data for Waves 1 to 3, Waves 1 to 2, and Waves 2 to 3, partly to see how consistent our findings were over the different periods considered.  Our later analyses used Wave 4 as well to allow us to study in detail sustained, delayed and temporary quitting, and to test consistency of our results for different periods (Waves 1 to 3, 2 to 4, and 1 to 4).  Note that you need data from at least three time points to study these forms of quitting.  Thus sustained quitting is YNN, delayed quitting is YYN- and temporary quitting is YNY, where Y (yes) indicates smoking and N (no) indicates not smoking.

 Dr. Kaplan says that there is no balance regarding the potential adverse effects of e-cigarettes which are a concern for many physicians. In response to this we would point out that the introduction to the paper did already mention in general terms the various possible upsides and downsides of e-cigarettes. However, we have slightly extended the initial sentence to mention some of the evidence supporting the view that any effects of e-cigarettes on health are substantially less than those of smoking.  As the paper is specifically about the relationship of e-cigarette use to quitting cigarettes, we would rather not, in a paper that is already rather long, to attempt to summarize all the evidence relating to health effects, let alone the other areas that we refer to in the first paragraph, such as on initiation and cigarette consumption.

 The final point of Dr. Kaplan was that we did not consider pharmacotherapeutic smoking cessation options other than e-cigarettes as possible confounders. However, we did consider them – please see the inclusion of “Ever used Chantix, varenicline or buproprion (Wellbutrin, Zyban)” in Tables 1 and 3.

 We hope that our paper can now be approved.

# Peer review: Reviewer response for version 2

**DOI:** 10.5256/f1000research.54961.r97677

**Published:** 2021-11-24

**Authors:** Riccardo Polosa

**Affiliations:** 1 Center of Excellence for the Acceleration of HArm Reduction (CoEHAR), Department of Clinical and Experimental Medicine, University of Catania, Catania, Italy

This paper investigates whether switching to e-cigarette use causes people to stop smoking, using data from the nationally representative PATH study. Significant elevated quit rates were reported. While there are identifiable imperfections in this study there are no apparent fatal flaws. If the entire literature on the topic looked like this, we would be fairly well informed. I have the following suggestions for improvement:

 There are several causal pathways at work here and the authors appear to have covered most angles accounting for several potential confounding variables. However, the authors should reassure the readers that their multivariate models included variables that would be sufficient to calculate propensity to start or quit smoking.

 The authors should make clear if the population of current smokers at baseline excludes those who have previously tried and failed e-cigarettes prior to enrollment as this “hard core” population may have an impact on subsequent quit rates. Anyone who already tried e-cigarettes and is still smoking is less likely to quit over a given period than people who still smoke but have never tried e-cigarettes. This is what happens when you remove most success stories from the study population. Moreover, it can be speculated that many of those who were inclined to switch to vaping would have already done so prior to enrollment, creating a stock-flow bias. These possibilities should be discussed in the text.

 At the individual level, the counterfactual of primary interest is whether someone successfully quits smoking because vaping exists, when either they would have failed to quit as they did without the aid of vaping *or* never would have even made the attempt but for trying vaping. I am not sure this has been addressed in the paper – e.g. by comparing at-risk people (people who smoke, and thus are at-risk for quitting) who have the exposure (which might be defined as trying vaping or some greater level of usage) to those who do not, trying to control for confounding.

 Details of vaping behavior (e.g. daily use at follow-up) may be important predictors of elevated quit rates. Those who vaped daily may considerably more likely to be smoking abstinent than average, while those who used them less-than-daily were a bit less likely. It would be interesting to stratify the analysis separating daily vs occasional (i.e. less-than-daily) e-cigarette use at follow up to see if there is a “dose/exposure-related” effect on the observed quit rates.

 The most compelling evidence that vaping causes *some* smoking cessation, though it does not allow for quantification, is the countless individual testimonials of quitting with vaping, including many accidental quitter stories and stories of having abandoned hope of quitting until vaping became an option. This evidence should be addressed, even as background.

 Some important papers are not cited here, e.g. Gomajee *et al.*, 2019, in which the authors looked at a large (the data included over 5000 people who smoke and 2000 who did formerly) nationally representative cohort in France, with recruitment starting in 2012 and following participants for an average of two years at the time their results were reported. Data was collected on not just recent e-cigarette use but also on when someone started vaping regularly, a simple useful question that is woefully missing from most studies.

 The possibility of self-selection bias for the PATH study must be acknowledged in the text.

 To the authors’ credit, they do not delve into offhand speculation about policy implications or statements of personal opinions, and are refreshingly epistemically modest. However, they should discuss how their time- and country-specific behavioral data might or might not generalize outside US, and suggest potential implications of their findings.

Is the work clearly and accurately presented and does it cite the current literature?

Yes

If applicable, is the statistical analysis and its interpretation appropriate?

I cannot comment. A qualified statistician is required.

Are all the source data underlying the results available to ensure full reproducibility?

Yes

Is the study design appropriate and is the work technically sound?

Yes

Are the conclusions drawn adequately supported by the results?

Yes

Are sufficient details of methods and analysis provided to allow replication by others?

Yes

Reviewer Expertise:

NA

I confirm that I have read this submission and believe that I have an appropriate level of expertise to confirm that it is of an acceptable scientific standard, however I have significant reservations, as outlined above.

# Response

**Published:** 2022-06-10

**Authors:** Peter Lee

**Affiliations:** P.N.Lee Statistics and Computing Ltd, UK

We thank Dr. Polosa for his comments.

 Dr. Polosa notes that we should reassure readers that our multivariate models include variables sufficient to calculate propensity to start or quit smoking.

 We note that our original discussion section already included a paragraph starting “The strengths of our work...” which included, as one of the strengths “analyses which take account of a very large numbers of other predictors of quitting”.  We did not consider propensity to start smoking, as the paper was about quitting. Though, of course, there might be some lurking relevant variable ignored, the list of predictors of quitting considered in our Table1 included as many as 55 variables.

 In his next paragraph, Dr. Polosa asks that we make clear if the population of current smokers at baseline excludes those previously trying and failing e-cigarettes prior to enrolment.

 In response to this we note that the fourth paragraph of the methods section defines what is meant by a current, former, or ever e-cigarette user, and that while our main model (results shown in Tables 2 and 3) relate to the relationship of quitting to ever e-cigarette use at baseline, sensitivity analysis 2 (results shown in Table 4) relate quitting to current e-cigarette use at baseline. Although there may be reasons, as Dr. Polosa states, why restricting attention to current e-cigarette users at baseline might show a stronger relationship, in practice there was no significant difference between the odds ratios for the main model and sensitivity analysis 2, both showing a positive relationship between e-cigarette use and quitting, as mentioned near the end of the first paragraph of the discussion.

 In the following paragraph (starting “At the individual level…”) Dr. Polosa states that we might compare quitting rates in people who smoke who have the exposure (e-cigarettes) and who do not have it, trying to control for confounding. But that is exactly what we have done – for example in Table 3, the odds ratio for “ever regularly used e-cigarette = yes” are based on a comparison with “ever regularly used e-cigarette = no” and are adjusted for the confounders described.

 In the next paragraph (starting “Details of vaping behavior…”) Dr. Polosa suggests that dose-response analyses relating quit rates to “dose” of e-cigarette use would be of interest.  We agree, but given the extent of the analyses already presented, and the fact that new waves of PHIM are by now available, we would prefer to carry out such analyses for a possible later publication. The possibility of this is now mentioned in the discussion in the paragraph starting “While it is clear that even more analyses could be run…”, where we have added the extra sentence “Further analyses could, for example, relate the probability of quitting to the extent of e-cigarette use, not considered in our analyses.”

 The following paragraph (starting “The most compelling evidence…”) refers to “countless individual testimonials of quitting with vaping…”. Without any reliable reference to this, we would not be able to say anything scientifically useful.  We wonder whether those who try vaping to quit and find it didn’t help report this as often as those who find it did work. Given the PATH study has no data here, we prefer not to go down this path.   

 Next, Dr. Polosa refers to some important papers not cited here, mentioning the paper by Gomajee et al., 2019. We have now included that paper in the reference list supporting the statement that “it is clear that by now there are quite a number of studies which report somewhat higher quit rates in those using e-cigarettes” in the fourth paragraph of the introduction.  However, please note that that list and the one following it are only a selection of papers finding or not finding a clear association, as noted by the “e.g.” at the beginning of each list.

 As regards raising the possibility of self-selection bias for the PATH study, we have modified the start of the paragraph in the discussion to read “The strengths of our work include the use of a prospective study design based on a study population which is reasonably representative of the US (though subject to some selection bias)…”.

 Finally, Dr. Polosa suggests that we discuss how our results might generalize outside the US. Here, without going into a detailed discussion covering all the major e-cigarette-using countries other than the US, we have just extended the conclusions section so that the final sentence now reads “The results seem consistent with most of the evidence from clinical trials, other analyses of the PATH study, and other epidemiological studies, and seem likely to apply to other countries and time periods.”

 We hope that our paper can now be approved.

# Peer review: Reviewer response for version 2

**DOI:** 10.5256/f1000research.54961.r81318

**Published:** 2021-03-29

**Authors:** John P. Pierce, Ruifeng Chen, Tarik Benmarhnia

**Affiliations:** 1 Division of Biostatistics, University of California, San Diego, San Diego, CA, USA; 2 Division of Epidemiology. Herbert Wertheim School of Public Health and Human Longevity Science, University of California San Diego, San Diego, CA, USA; 3 Scripps Institution of Oceanography, University of California San Diego, San Diego, CA, USA; 4 Moores Cancer Center, Herbert Wertheim School of Public Health and Human Longevity Science, University of California, San Diego, San Diego, CA, USA

This paper has been rewritten to address concerns that were expressed with the initial version.

- **Previous comment: The introduction presents a somewhat limited review of the literature.**

***Authors’ Response***: dismissed this comment, asserting that their presentation was a relatively succinct summary of the findings in the literature.

***Reviewer 2 ^nd^ response:***

 The National Academies of Science Engineering and Medicine ^1^ highlighted that the research addressed at least two different research questions. The first was that e-cigarettes with nicotine were more effective than e-cigarettes without nicotine in medium term abstinence after a quit attempt. The second is the effectiveness of e-cigarettes as cessation aids compared to any other method. It is this question that is the focus of this paper and the NASEM report noted that, as of 2018, RCT’s had not really addressed this question

 The authors now reference 10 randomized trials (RCTs) most of which do not address the research question that they are addressing in this paper. 

 Further, there are a number of different “systematic” reviews of this literature and they differ widely in their conclusions. Rather than present both sides, the authors “cherry-pick” from these reviews to support their hypothesis.

- **Previous comment on the importance of limiting consideration to people who had made a quit attempt. **This previous comment noted that the likelihood of a quit attempt was much higher in e-cigarette users in the PATH study (many who started using e-cigarettes did so because they were trying to quit) than it was in the control population. Thus, the higher probability of making a quit attempt could explain the difference in smoking cessation and so the difference should not be attributed to the use of e-cigarettes.

***Authors’ response*** - they have adjusted for quitting in some analyses but “ *We did not say that the population needed to have made a recent quit attempt, as this was not a requirement in our study. Our analyses compared quit rates between e-cigarette users and non-users to try to answer the simpler, and highly relevant, question ‘are e-cigarette users more likely to quit?’*

**Reviewer 2 ^nd^ response**:  Furthering our previous comment, there now are multiple publications demonstrating that e-cigarettes are the most popular choice for smokers looking for help during a quit attempt. In other words, those who make quit attempt are more likely to use e-cigarettes than those who don’t make quit attempt, thus, to answer the authors “simpler” question - of course e-cigarette users are more likely to quit, because the reason they are using an e-cigarette is to help them quit. More generally speaking, this is an example of Simpson’s paradox. The effects of e-cigarette use are totally different among those who make quit attempt and those who don’t. The importance of not making this error in analyzing cohort studies was not originally brought up by our team. Rather, it was a warning included in the National Academies of Science, Engineering and Medicine 2018 report on e-cigarettes.

 We noticed that the authors have made a minimum attempt to adjust for this confounder variable by using “Have tried to quit completely”. This is not sufficient to address the problem. Firstly, the interaction between e-cigarette use and the quit attempts has been shown to be important in previous studies (those focused on recent quit attempters). Second, the variable “Have tried to quit completely” is not qualified by time in any way. Thus, the attempt to quit completely may have happened in the past year but the use of e-cigarettes may have happened before then. Thus, the exposure might have happened before the assessment of the confounder, a classical error for anyone studying an etiological problem. By refusing to separate their consideration to smokers who had made a quit attempt and who hadn’t, the authors have introduced a substantial bias into their analysis that invalidates their findings

- **Is it appropriate to use an analytic strategy based on stepwise regressions to select model covariates that are then use to infer causal relationships between e-cigarettes and quitting outcomes.**

The authors main research question of interest is etiological (as opposed to one that seeks simply to describe the data or to address a predictive research questions (see Hernán 2018, Hernán, Hsu *et al*. 2019) ^2^^,^^3^.  For an etiological question, the identification and conditioning on potential confounders is crucial. For such questions, selecting potential confounders in an outcome model (like a multivariable model) based on statistical criteria has been repeatedly shown to be erroneous (Greenland 1989, Smith 2018) ^4^^,^^5^ especially when covariates induced by the exposure and  the outcome are included which may lead to collider-stratification bias. Therefore, the entire analytical plan has to be revisited as all effect estimates presented are based on an erroneous analytical approach and none of the ORs can be used to infer any relationship between e-cigarette use and cigarettes quitting.

Is the work clearly and accurately presented and does it cite the current literature?

Partly

If applicable, is the statistical analysis and its interpretation appropriate?

Partly

Are all the source data underlying the results available to ensure full reproducibility?

Yes

Is the study design appropriate and is the work technically sound?

No

Are the conclusions drawn adequately supported by the results?

No

Are sufficient details of methods and analysis provided to allow replication by others?

Yes

Reviewer Expertise:

Behavioral Epidemiology, with a major focus on tobacco use behavior.

We confirm that we have read this submission and believe that we have an appropriate level of expertise to state that we do not consider it to be of an acceptable scientific standard, for reasons outlined above.

## References

[1] : Public Health Consequences of E-Cigarettes.2018; 10.17226/24952 10.17226/24952 29894118

[2] : The C-Word: Scientific Euphemisms Do Not Improve Causal Inference From Observational Data. *Am J Public Health* .108(5) : 10.2105/AJPH.2018.304337 616-619 10.2105/AJPH.2018.304337 29565659PMC5888052

[3] : A Second Chance to Get Causal Inference Right: A Classification of Data Science Tasks. *CHANCE* .2019;32(1) : 10.1080/09332480.2019.1579578 42-49 10.1080/09332480.2019.1579578

[4] : Modeling and variable selection in epidemiologic analysis. *Am J Public Health* .1989;79(3) : 10.2105/ajph.79.3.340 340-9 10.2105/ajph.79.3.340 2916724PMC1349563

[5] : Step away from stepwise. *Journal of Big Data* .2018;5(1) : 10.1186/s40537-018-0143-6 10.1186/s40537-018-0143-6

# Response

**Published:** 2021-04-08

**Authors:** Peter Lee

**Affiliations:** P.N.Lee Statistics and Computing Ltd, UK

We thank Drs Chen and Pierce for their second set of comments. Our replies, disagreeing with virtually all the points they make, are given below italics. We do not intend to revise our paper further based on this second set of comments.

 Not Approved

 This paper has been rewritten to address concerns that were expressed with the initial version.

- **Previous comment: The introduction presents a somewhat limited review of the literature.**

***Authors’ Response***: dismissed this comment, asserting that their presentation was a relatively succinct summary of the findings in the literature.

***Reviewer 2 ^nd^ response:***

 The National Academies of Science Engineering and Medicine ^1^ highlighted that the research addressed at least two different research questions. The first was that e-cigarettes with nicotine were more effective than e-cigarettes without nicotine in medium term abstinence after a quit attempt.

*This first question is not particularly relevant to our paper, which is concerned with comparing quit rates in e-cigarette users as a whole versus non-users of e-cigarettes*.

 The second is the effectiveness of e-cigarettes as cessation aids compared to any other method. It is this question that is the focus of this paper

*We don’t understand why Drs Chen and Pierce think we were attempting to answer this second question?  We weren’t.*

 and the NASEM report noted that, as of 2018, RCT’s had not really addressed this question

 The authors now reference 10 randomized trials (RCTs) most of which do not address the research question that they are addressing in this paper. 

 Further, there are a number of different “systematic” reviews of this literature and they differ widely in their conclusions. Rather than present both sides, the authors “cherry-pick” from these reviews to support their hypothesis.

*Drs Chen and Pierce are commenting on the paragraph in the introduction of our paper concerning randomized controlled trials. This was intended to give some context to our paper, though of course our main analyses concern evidence from a non-randomized epidemiological study.  we had cited three references as suggesting that the evidence from randomized controlled trials was consistent with higher quit rates in those using e-cigarettes, one of these being a recent prestigious cochran report. While we had selected these three major reviews based on a look at the recent literature, there was certainly no intention to “cherry-pick”, as we are accused of.  Looking back at the reviews currently available, we could cite other reviews that consider that the evidence suggests (as it does) higher quit rates in e-cigarette users, but none that it suggest lower quit rates. We consider our paragraph to be a fair reflection of the evidence from rcts*.

**Previous comment on the importance of limiting consideration to people who had made a quit attempt. **This previous comment noted that the likelihood of a quit attempt was much higher in e-cigarette users in the PATH study (many who started using e-cigarettes did so because they were trying to quit) than it was in the control population. Thus, the higher probability of making a quit attempt could explain the difference in smoking cessation and so the difference should not be attributed to the use of e-cigarettes.

***Authors’ response*** - they have adjusted for quitting in some analyses but “ *We did not say that the population needed to have made a recent quit attempt, as this was not a requirement in our study. Our analyses compared quit rates between e-cigarette users and non-users to try to answer the simpler, and highly relevant, question ‘are e-cigarette users more likely to quit?’*

**Reviewer 2 ^nd^ response**:  Furthering our previous comment, there now are multiple publications demonstrating that e-cigarettes are the most popular choice for smokers looking for help during a quit attempt. In other words, those who make quit attempt are more likely to use e-cigarettes than those who don’t make quit attempt, thus, to answer the authors “simpler” question - of course e-cigarette users are more likely to quit, because the reason they are using an e-cigarette is to help them quit. More generally speaking, this is an example of Simpson’s paradox. The effects of e-cigarette use are totally different among those who make quit attempt and those who don’t. The importance of not making this error in analyzing cohort studies was not originally brought up by our team. Rather, it was a warning included in the National Academies of Science, Engineering and Medicine 2018 report on e-cigarettes.

 We noticed that the authors have made a minimum attempt to adjust for this confounder variable by using “Have tried to quit completely”. This is not sufficient to address the problem. Firstly, the interaction between e-cigarette use and the quit attempts has been shown to be important in previous studies (those focused on recent quit attempters). Second, the variable “Have tried to quit completely” is not qualified by time in any way. Thus, the attempt to quit completely may have happened in the past year but the use of e-cigarettes may have happened before then. Thus, the exposure might have happened before the assessment of the confounder, a classical error for anyone studying an etiological problem. By refusing to separate their consideration to smokers who had made a quit attempt and who hadn’t, the authors have introduced a substantial bias into their analysis that invalidates their findings.

*In using the word “refusing” in their final sentence, Chen and Pierce seem to have missed completely the highly relevant fact that in our analyses based on waves 1 to 4 we included a sensitivity analysis 6 excluding those not attempting to quit, and that for sustained quitting (perhaps the most relevant measure) the associations were weaker if anything than those seen in the main analysis, indicating that increased quitting rates were seen also in those not attempting to quit.*

**Is it appropriate to use an analytic strategy based on stepwise regressions to select model covariates that are then use to infer causal relationships between e-cigarettes and quitting outcomes.**

 The authors main research question of interest is etiological (as opposed to one that seeks simply to describe the data or to address a predictive research questions (see Hernán 2018, Hernán, Hsu *et al*. 2019) ^2,3^.  For an etiological question, the identification and conditioning on potential confounders is crucial. For such questions, selecting potential confounders in an outcome model (like a multivariable model) based on statistical criteria has been repeatedly shown to be erroneous (Greenland 1989, Smith 2018) ^4,5^ especially when covariates induced by the exposure and  the outcome are included which may lead to collider-stratification bias. Therefore, the entire analytical plan has to be revisited as all effect estimates presented are based on an erroneous analytical approach and none of the ORs can be used to infer any relationship between e-cigarette use and cigarettes quitting.

*While we agree that in principle one shouldn’t adjust for variables induced by the exposure and the outcome, I do not believe that we had done so here. The variables we adjusted for were generally characteristics that were present before take up of e-cig use and before quitting, and therefore would not cause problems with over-correction*.

**References**

 1. Committee on the Review of the Health Effects of Electronic Nicotine Delivery Systems, Board on Population Health and Public Health Practice, Health and Medicine Division, National Academies of Sciences, Engineering, and Medicine: Public Health Consequences of E-Cigarettes. 2018. Publisher Full Text

 2. Hernán MA: The C-Word: Scientific Euphemisms Do Not Improve Causal Inference From Observational Data. *Am J Public Health*. **108** (5): 616-619 PubMed Abstract | Publisher Full Text

 3. Hernán M, Hsu J, Healy B: A Second Chance to Get Causal Inference Right: A Classification of Data Science Tasks. *CHANCE*. 2019; **32** (1): 42-49 Publisher Full Text

 4. Greenland S: Modeling and variable selection in epidemiologic analysis. *Am J Public Health*. 1989; **79** (3): 340-9 PubMed Abstract | Publisher Full Text

 5. Smith G: Step away from stepwise. *Journal of Big Data*. 2018; **5** (1). Publisher Full Text

# Peer review: Reviewer response for version 1

**DOI:** 10.5256/f1000research.28877.r70981

**Published:** 2020-09-14

**Authors:** John P. Pierce, Ruifeng Chen

**Affiliations:** 1 Division of Biostatistics, University of California, San Diego, San Diego, CA, USA; 2 Moores Cancer Center, Herbert Wertheim School of Public Health and Human Longevity Science, University of California, San Diego, San Diego, CA, USA

In the background of the abstract, the authors state that their goal is to address the research question whether e-cigarettes encourage smokers to quit using a quality nationally representative cohort study from the United States.

 The introduction presents a somewhat limited review of the literature. In the discussion of the randomized trials the authors infer that these trials reached statistically significant findings where e-cigarettes were favored. This is a misrepresentation of the literature.

 The authors note the following problems that have been identified with observational studies addressing the target research question: studies in which the exposure measure does not precede the outcome measure (as happens in cross-sectional studies), the use of non-representative populations, and failure to fully account for the many potential confounding factors. While these are appropriate criticisms of some of the literature, they are far from complete. For example, in 2018, the US National Academies of Science, Engineering and Medicine put out a major report on e-cigarettes where they expressed concern about the quality of some of the papers addressing whether these products were cessation aids: In addition to these three concerns, this report noted the importance of the study focusing on quit attempts with a comparable control group – this issue has been highlighted in the 2019 paper by Pierce *et al.* in Nicotine and Tobacco Research ^1^. This analysis showed that the likelihood of a quit attempt was much higher in e-cigarette users in the PATH study (many who started using e-cigarettes did so because they were trying to quit) than in was in the control population. Thus, the higher probability of making a quit attempt could explain the difference in smoking cessation and so the difference should not be attributed to the use of e-cigarettes.

 However, the authors go further and argue “ *Problems involve the …failure to limit attention to established e-cigarette users,”* (Para 4 introduction). It is not clear what the authors mean by this? Do they mean that the study population need to have developed a consistent pattern of dual use of cigarettes and e-cigarettes? What is their justification for such a limitation when addressing the stated research question? It is apparent that they do not place such a limitation on their own analyses.

 Indeed, the authors have not laid out specific research questions or hypotheses that guide the analyses that they undertake in this paper. This is an important omission.

 In the methods, the authors state that analyses are based on individuals with relevant data available at Waves 1, 2 and 3 on smoking and e-cigarette use. They do not mention that the population needed to have made a recent quit attempt. As noted above, unless they demonstrate that the populations that they choose are comparable on this variable, then their study is confounded.

 The methods are not at all clear about the outcome measure used in this analysis. In their section outlining their main analyses, the authors use the term “quitting during follow-up”. What does this mean? A logical interpretation is that a quit attempt was made in the year prior to the follow-up survey. However, the authors have not addressed the quit attempt data in the methods. Do they use a point prevalence of former smoking at the follow-up survey? If so, how can this be described as successful smoking cessation? This is one of the most critical points in the paper. Most other papers discussing successful quitting require abstinence for either 6 of 12 months at follow-up (see, Gilpin *et al*. 1997 ^2^). This is particularly important in an observational study when a person quit at follow-up could have quit only for the day before the survey, for example.

 The potential confounders in this analysis include those variables that are associated with use of e-cigarettes as well as variables that are associated with successful cessation. The authors omit any discussion of variables associated with the probability of a smoker using an e-cigarette, thus seriously confounding their analyses. At least in a supplement, there should be a summary table of these predictors outlining how they are associated with e-cigarette use.

 Analytic Plan: The analysis plan lays out a stepwise forward selection logistic regression, adjusted for selected covariates. What is unclear is how the authors use step 5 & 6 of their analytic plan in drawing their inferences.

 There is a problem with the use of study weights. For their Wave 2 to Wave 3 analysis, it would seem that the appropriate weights would be the Wave 2 weights,

 A figure laying out the different analyses with samples sizes (including loss to follow-up) would be very helpful to the reader.

 Results: The unadjusted quitting rates by ever use of e-cigarettes should be presented a in table in the results section.

 Also, the authors should report the percentage of subjects excluded in the multiple regressions.

 Any reworking of this manuscript should discuss the four recent analyses that address e-cigarettes and smoking using the PATH data. All these papers use appropriate analytic procedures and arrive at opposite conclusions to those of the authors ^3^^- ^^6^.

Is the work clearly and accurately presented and does it cite the current literature?

Partly

If applicable, is the statistical analysis and its interpretation appropriate?

Partly

Are all the source data underlying the results available to ensure full reproducibility?

Yes

Is the study design appropriate and is the work technically sound?

No

Are the conclusions drawn adequately supported by the results?

No

Are sufficient details of methods and analysis provided to allow replication by others?

Yes

Reviewer Expertise:

Behavioral Epidemiology, with a major focus on tobacco use behavior.

We confirm that we have read this submission and believe that we have an appropriate level of expertise to state that we do not consider it to be of an acceptable scientific standard, for reasons outlined above.

## References

[1] : A Source of Bias in Studies of E-Cigarettes and Smoking Cessation. *Nicotine & Tobacco Research* .2020;22(5) : 10.1093/ntr/ntz143 861-862 10.1093/ntr/ntz143 PMC717128031398246

[2] : Duration of smoking abstinence and success in quitting. *J Natl Cancer Inst* .1997;89(8) : 10.1093/jnci/89.8.572 572-6 10.1093/jnci/89.8.572 9106646

[3] : Role of e-cigarettes and pharmacotherapy during attempts to quit cigarette smoking: The PATH Study 2013-16. *PLoS One* .2020;15(9) : 10.1371/journal.pone.0237938 e0237938 10.1371/journal.pone.0237938 32877429PMC7467279

[4] : E-Cigarette Use to Aid Long-Term Smoking Cessation in the US: Prospective Evidence from the PATH Cohort Study. *Am J Epidemiol* .2020; 10.1093/aje/kwaa161 10.1093/aje/kwaa161 32715314PMC7705599

[5] : Association of electronic cigarette vaping and subsequent smoking relapse among former smokers. *Drug and Alcohol Dependence* .2019;199: 10.1016/j.drugalcdep.2019.01.043 10-17 10.1016/j.drugalcdep.2019.01.043 30978519PMC6743978

[6] : Association of Electronic Nicotine Delivery System Use With Cigarette Smoking Relapse Among Former Smokers in the United States. *JAMA Netw Open* .2020;3(6) : 10.1001/jamanetworkopen.2020.4813 e204813 10.1001/jamanetworkopen.2020.4813 32501492PMC7275247

# Response

**Published:** 2020-10-02

**Authors:** Peter Lee

**Affiliations:** P.N.Lee Statistics and Computing Ltd, UK

**We thank the reviewers for their comments which we attempt to answer in detail below.  Please note that, as advised by the journal, our proposed changes to the paper have not, at this stage, been made, pending comments from other reviewers on our paper.  Please also note that, as explained in more detail below, some of the points raised by the reviewers will be dealt with more fully in a further paper we are currently preparing based on data from Waves 1 to 4, as mentioned in the penultimate paragraph of the conclusions section.   As will be evident, the reviewers original comments are in normal type and our replies and suggested changes to the paper are in bold.  We hope that our reply and the changes will affect the reviewers’ opinion of our work.**

In the background of the abstract, the authors state that their goal is to address the research question whether e-cigarettes encourage smokers to quit using a quality nationally representative cohort study from the United States.

The introduction presents a somewhat limited review of the literature.

**The introduction was never intended to be a fully detailed and comprehensive review of the extensive literature relating e-cigarettes to quitting, the main purpose of the paper being to describe the results of our own analyses.  Nevertheless the introduction includes as many as 39 references, and we feel gives a relatively succinct summary of the findings from the literature available at the time of writing.**

In the discussion of the randomized trials the authors infer that these trials reached statistically significant findings where e-cigarettes were favored. This is a misrepresentation of the literature.

**We made no reference to statistical significance and had not intended to imply this.  We were merely summarizing the direction of the differences reported in the papers cited, virtually all of which indicated a higher quit rate in the nicotine e-cigarette group.  However to avoid confusion, the second sentence of the second paragraph of the discussion will be amended so that it ends**

**‘…generally indicates higher quit rates in the nicotine e-cigarette group, although not all the differences cited were statistically significant (at p<0.05), and one study did not find such higher quit rates** **(Halpern et al., 2018)** **.’**

The authors note the following problems that have been identified with observational studies addressing the target research question: studies in which the exposure measure does not precede the outcome measure (as happens in cross-sectional studies), the use of non-representative populations, and failure to fully account for the many potential confounding factors. While these are appropriate criticisms of some of the literature, they are far from complete. For example, in 2018, the US National Academies of Science, Engineering and Medicine put out a major report on e-cigarettes where they expressed concern about the quality of some of the papers addressing whether these products were cessation aids:

**We had already stated in the first sentence of paragraph 4 of the discussion that’…a recent review considered the evidence is inconclusive due to the low quality of the research’ citing** **Malas et al. (2016)** **.  We will amend this to start ‘…recent reviews have considered the evidence…’ and additionally cite the reference to the major report in 2018 that the reviewers mention.**

In addition to these three concerns, this report noted the importance of the study focusing on quit attempts with a comparable control group – this issue has been highlighted in the 2019 paper by Pierce *et al.* in Nicotine and Tobacco Research ^1^ .

**In the second sentence of paragraph 4 of the discussion we will add ‘the use of non-comparable control groups’ after ‘the use of unrepresentative populations’.**

This analysis showed that the likelihood of a quit attempt was much higher in e-cigarette users in the PATH study (many who started using e-cigarettes did so because they were trying to quit) than it was in the control population. Thus, the higher probability of making a quit attempt could explain the difference in smoking cessation and so the difference should not be attributed to the use of e-cigarettes.

**We already do adjust for aspects of smoking related to quitting in our analyses, as shown in Tables 1 and 3.**

However, the authors go further and argue “ *Problems involve the …failure to limit attention to established e-cigarette users,”* (Para 4 introduction). It is not clear what the authors mean by this? Do they mean that the study population need to have developed a consistent pattern of dual use of cigarettes and e-cigarettes? What is their justification for such a limitation when addressing the stated research question? It is apparent that they do not place such a limitation on their own analyses.

**It does not seem sensible to include among e-cigarette users those who have in the past tried them once or twice and abandoned them as they did not like them.  The reviewers state that we did not limit attention to established users.  This is untrue; that we do limit attention to established users is very clearly stated in the third paragraph of the methods section.**

Indeed, the authors have not laid out specific research questions or hypotheses that guide the analyses that they undertake in this paper. This is an important omission.

**In the introduction, the first sentence of the final paragraph is ‘Here we describe results from a prospective study aimed at avoiding such weaknesses.’ which might be regarded as a statement of our objectives.  However we will insert a sentence after this to read:**

**‘The main objective of our analyses is to quantify the relationship between e-cigarette use in smokers and subsequent cessation of smoking, with detailed adjustment for the multitude of factors that may differ between e-cigarette users and non-users.’  We will also amend the next sentence to start ‘Our analyses are based on’ rather than ‘It is based on…’.**

In the methods, the authors state that analyses are based on individuals with relevant data available at Waves 1, 2 and 3 on smoking and e-cigarette use. They do not mention that the population needed to have made a recent quit attempt. As noted above, unless they demonstrate that the populations that they choose are comparable on this variable, then their study is confounded.

**We did not say that the population needed to have made a recent quit attempt, as this was not a requirement in our study, and indeed was not a requirement of a number of the other studies we had cited in our discussion** **(Berry et al., 2019; Kalkhoran et al., 2019; Verplaetse et al., 2019)** **, though it was in others** **(Benmarhnia et al., 2018; Watkins et al., 2019)** **.  Those who used e-cigarettes at baseline may not at that time have used them intending to quit, but may have found during follow-up that they could meet their nicotine needs without smoking cigarettes.  Our analyses compared quit rates between e-cigarette users and non-users to try to answer the simpler, and highly relevant, question ‘are e-cigarette users more likely to quit?’  We prefer to present results based on the whole population of baseline smokers, thus using a larger sample size than if we restricted attention to quit attempters,  and to try to avoid bias by taking into account a range of predictor variables related to quitting.**

**However we will add a second paragraph in the methods section as follows.**

**‘Some studies of data from Waves 1 to 3 of the PATH study** **(Benmarhnia et al., 2018; Pierce et al., 2020a; Watkins et al., 2019)** **have limited analyses to quit attempters, but others** **(Berry et al., 2019; Kalkhoran et al., 2019; Verplaetse et al., 2019)** **have not.  Although such a limitation more closely mimics randomized control trials** **(Pierce et al., 2020b)** **we prefer not to do so, and to avoid bias by adjusting for aspects of quitting in analyses.  Our approach uses a larger sample size and provides results that are more representative of the whole population of baseline smokers’.**

**As already noted, we intend to carry out some additional analyses limited to quit attempters in the paper being prepared based on data from Waves 1 to 4, though we do note for the reviewers’ information that a preliminary analysis we conducted, based on data from Waves 1 and 2 and limiting attention to those who at Wave 2 had ever made a quit attempt, produced an adjusted odds ratio of 1.20 (95%CI 0.91-1.57) which is very similar to the estimate we give in Table 2 of our paper of 1.23 (0.94-1.61).**

The methods are not at all clear about the outcome measure used in this analysis. In their section outlining their main analyses, the authors use the term “quitting during follow-up”. What does this mean? A logical interpretation is that a quit attempt was made in the year prior to the follow-up survey. However, the authors have not addressed the quit attempt data in the methods. Do they use a point prevalence of former smoking at the follow-up survey? If so, how can this be described as successful smoking cessation? This is one of the most critical points in the paper. Most other papers discussing successful quitting require abstinence for either 6 of 12 months at follow-up (see, Gilpin *et al*. 1997 ^2^ ). This is particularly important in an observational study when a person quit at follow-up could have quit only for the day before the survey, for example.

**We certainly thought that we had made it clear enough in the methods that we were defining smoking status as at each Wave, and defining quitting based on being a current established smoker at baseline and a former established smoker at follow-up.  While we agree that some former smokers may only have quit for a relatively short time, we believe that our method still provides meaningful results.  However, we will extend the first sentence of the old fourth paragraph of the methods to read**

**‘The main analysis for each period relates ever e-cigarette use at baseline to the probability of being an established former smoker at follow-up (referred to subsequently as either “quitting” or “quitting during follow-up”), with adjustment for predictor variables measured at baseline.’**

**Again, for the reviewers’ information, we repeated our Wave 1 to 2 analysis, redefining quitting as having quit for at least 30 days.  This produced an adjusted estimate of 1.26 (95%CI 0.93-1.70) which is very similar to the estimate we give in Table 2 of our paper of 1.23 (0.94-1.61).**

The potential confounders in this analysis include those variables that are associated with use of e-cigarettes as well as variables that are associated with successful cessation. The authors omit any discussion of variables associated with the probability of a smoker using an e-cigarette, thus seriously confounding their analyses. At least in a supplement, there should be a summary table of these predictors outlining how they are associated with e-cigarette use.

**We will extend the fifth paragraph of the methods section to read as follows:**

**‘The specific predictor variables are listed in the Results section, with fuller details of their definition given in the *Extended data.  *While the variables were chosen as being suggested by the literature as being related to smoking, the *Extended data *also provides information, based on Wave 1, of their association with ever e-cigarette use.  As shown there, ever use was highly significantly (p < 0.001) more frequent in the young and in females, and after adjustment for age and sex, was also highly significantly related to a range of the predictor variables considered, being less frequent in Hispanics and Blacks, and more frequent in those with more income or education, those who ever use other tobacco products, those who have a perceived greater need for tobacco, those who have tried to quit more often, those who plan to quit, those who find it hard to stop smoking and those who have used quitting aids.  Users were also clearly more likely to have significant problems more recently with sleeping, anxiety and distress, to see a doctor more often, to use the internet often, and to use various different types of drugs (but not cocaine or crack).  At most weak relationships were seen with smoking by family and friends, awareness of the hazards of smoking, use of alcohol, body mass index, or self-perception of physical health or quality of life.  Little relationship was also  seen between ever e-cigarette use and daily cigarette consumption, a finding which was reported earlier** **(Lee et al., 2020)** **, where it was suggested that it was explained by smokers taking up e-cigarettes having higher consumption initially, reduced by partial replacement of cigarettes by e-cigarettes.’**

**We will also, in due course, add to the Extended data a section describing and presenting the output from which the above results are summarized.**

Analytic Plan: The analysis plan lays out a stepwise forward selection logistic regression, adjusted for selected covariates. What is unclear is how the authors use step 5 & 6 of their analytic plan in drawing their inferences.

**Our inferences are drawn from the results of the step 5 and 6 analyses which, reassuringly, produced the same models.  The results in Table 2 compare unadjusted odds ratios with those adjusted for the final (step 5/6) models shown in Table 3.**

There is a problem with the use of study weights. For their Wave 2 to Wave 3 analysis, it would seem that the appropriate weights would be the Wave 2 weights,

**In fact we did use Wave 2 weights for the Wave 2 to Wave 3 analysis.  We will correct the end of the second sentence of the first paragraph of the Methods section to read**

**‘, and take account of the person-based weights of the baseline population’.**

A figure laying out the different analyses with samples sizes (including loss to follow-up) would be very helpful to the reader.

Results: The unadjusted quitting rates by ever use of e-cigarettes should be presented in a table in the results section.

Also, the authors should report the percentage of subjects excluded in the multiple regressions.

**We already present in Tables 1 and 2 sample sizes in each of the main analyses.  We will add information on the proportion of the baseline population that were not followed up, but do not think that this merits a figure.  We will also extend Table 2 to include additional lines to cover the other points made by the reviewers.  Thus the revised tables will start with the following lines:**

**Table 1.**

**Table 2.**

Any reworking of this manuscript should discuss the four recent analyses that address e-cigarettes and smoking using the PATH data. All these papers use appropriate analytic procedures and arrive at opposite conclusions to those of the authors ^3^ ^-^ ^6^ .

**Of the four references which the reviewers cite, two** **(Dai and Leventhal, 2019; Everard et al., 2020)** **relate to relapse, which is outside the scope of the paper, and not quitting, and one of these** **(Dai and Leventhal, 2019)** **in any case states that ‘Baseline e-cigarette use was not associated with smoking relapse at follow-up after covariate adjustment.’  We do not propose to cite these two references, as our paper concerns quitting.**

**One of the other two references the reviewers cite** **(Pierce et al., 2020a)** **, not published at the time our paper was prepared, describes analyses based on Waves 1 to 3 of the PATH study.  We have, in the discussion in paragraphs 3 to 7, already summarized findings from five other analyses based on the PATH study which are consistent with e-cigarette use increasing the probability of quitting cigarettes.  To take into account this paper, and to provide further detail in the discussion, we propose to make the following two changes.**

**First, we will extend the second paragraph of the discussion to read as follows:**

**‘Six other related analyses based on the first three Waves 1 of the PATH study have previously been published.  The first five analyses summarized below** **(Benmarhnia et al., 2018; Berry et al., 2019; Kalkhoran et al., 2019; Verplaetse et al., 2019; Watkins et al., 2019)** **are consistent with e-cigarette use increasing the probability of quitting cigarettes, despite variation in whether Wave 3 data has been used or not, whether analyses are restricted to those attempting quitting at baseline, the definition of abstinence used, the confounding variables adjusted for, and other analytical details.  However, the final analysis** **(Pierce et al., 2020a)** **only reported a small and non-significant increase in quitting related to e-cigarette use.’**

**Second, we will add a new paragraph about the latest study at the end of the paragraphs describing the results of the first five analyses as follows:**

**‘A third analysis based on data from Waves 1, 2 and 3** **(Pierce et al., 2020a)** **restricted attention to adult (ages 18+) smokers identified at Wave 1 who reported a quit attempt before Wave 2 and completed Wave 3.  12 month abstinence at Wave 3 among e-cigarette users was slightly but non-significantly reduced as compared both to users of pharmacotherapy to quit or no product.’**

**The other reference cited by the reviewers** **(Chen et al., 2020)** **refers to analyses based on Waves 1 to 4 of the PATH study.  Partly because our analyses relate to Waves 1 to 3, and partly because we are currently developing analyses based on Waves 1 to 4 for a further publication, we strongly prefer not to consider analyses that include Wave 4 data in our discussion.**

**We will also add two extra sentences at the end of the penultimate paragraph of our discussion section, which refers to the planned further paper, as follows:**

**‘That paper might also consider different definitions of quitting, such as at least 30 day quitting, and investigate the effect of restricting attention to those attempting quitting at baseline.  It will also describe and comment on other publications that have used data up to Wave 4’.**

Is the work clearly and accurately presented and does it cite the current literature?

Partly  

**It was never intended to consider literature based on analyses using PATH Wave 4 data, or considering relapse, and the huge literature on e-cigarettes from other studies is covered in fair detail.**

< >Is the study design appropriate and is the work technically sound?Are sufficient details of methods and analysis provided to allow replication by others?If applicable, is the statistical analysis and its interpretation appropriate? Are all the source data underlying the results available to ensure full reproducibility?Are the conclusions drawn adequately supported by the results?

We confirm that we have read this submission and believe that we have an appropriate level of expertise to state that we do not consider it to be of an acceptable scientific standard, for reasons outlined above.

**We hope that our submission, when the revisions described are made, will now be considered of an acceptable scientific standard.**

References

Benmarhnia, T., Pierce, J.P., Leas, E., White, M.M., Strong, D.R., Noble, M.L., Trinidad, D.R., 2018. Can e-cigarettes and pharmaceutical aids increase smoking cessation and reduce cigarette consumption? Findings from a nationally representative cohort of American smokers. Am. J. Epidemiol. 187, 11, 2397-2404. DOI:10.1093/aje/kwy129.

Berry, K.M., Reynolds, L.M., Collins, J.M., Siegel, M.B., Fetterman, J.L., Hamburg, N.M., Bhatnagar, A., Benjamin, E.J., Stokes, A., 2019. E-cigarette initiation and associated changes in smoking cessation and reduction: the Population Assessment of Tobacco and Health Study, 2013-2015. Tob. Control. 28, 1, 42-49. DOI:10.1136/tobaccocontrol-2017-054108.

Chen, R., Pierce, J.P., Leas, E.C., White, M.M., Kealey, S., Strong, D.R., Trinidad, D.R., Benmarhnia, T., Messer, K., 2020. E-cigarette use to aid long-term smoking cessation in the US: Prospective evidence from the PATH Cohort Study. Am. J. Epidemiol. Published online ahead of print July 27, 2020 (doi: 10.1093/aje/kwaa161). DOI:10.1093/aje/kwaa161. Erratum appears in Am J Epidemiol. 2020 Sep 12;kwaa193. doi: 10.1093/aje/kwaa193.

Dai, H., Leventhal, A.M., 2019. Association of electronic cigarette vaping and subsequent smoking relapse among former smokers. Drug Alcohol Depend. 199, 10-17. DOI:10.1016/j.drugalcdep.2019.01.043.

Everard, C.D., Silveira, M.L., Kimmel, H.L., Marshall, D., Blanco, C., Compton, W.M., 2020. Association of electronic nicotine delivery system use with cigarette smoking relapse among former smokers in the United States. JAMA Netw. Open. 3, 6, e204813. DOI:10.1001/jamanetworkopen.2020.4813.

Halpern, S.D., Harhay, M.O., Saulsgiver, K., Brophy, C., Troxel, A.B., Volpp, K.G., 2018. A pragmatic trial of e-cigarettes, incentives, and drugs for smoking cessation. N. Engl. J. Med. 378, 24, 2302-2310. DOI:10.1056/NEJMsa1715757.

Kalkhoran, S., Chang, Y., Rigotti, N.A., 2019. E-cigarettes and smoking cessation in smokers with chronic conditions. Am. J. Prev. Med. 57, 6, 786-791. DOI:10.1016/j.amepre.2019.08.017.

Lee, P., Fry, J., Forey, B., Coombs, K.J., Thornton, A.J., 2020. Cigarette consumption in adult dual users of cigarettes and e-cigarettes: a review of the evidence, including new results from the PATH study [version 1; peer review: awaiting peer review] F1000Res. 9, 630. DOI:10.12688/f1000research.24589.1.

Malas, M., van der Tempel, J., Schwartz, R., Minichiello, A., Lightfoot, C., Noormohamed, A., Andrews, J., Zawertailo, L., Ferrence, R., 2016. Electronic cigarettes for smoking cessation: A systematic review. Nicotine Tob. Res. 18, 10, 1926-1936. DOI:10.1093/ntr/ntw119.

Pierce, J.P., et al., 2020a. Role of e-cigarettes and pharmacotherapy during attempts to quit cigarette smoking: The PATH Study 2013-16. PLoS One. 15, 9, e0237938. DOI:10.1371/journal.pone.0237938.

Pierce, J.P., Messer, K., Leas, E.C., Kealey, S., White, M.M., Benmarhnia, T., 2020b. A source of bias in studies of e-cigarettes and smoking cessation. Nicotine Tob. Res. 22, 5, 861-862. DOI:10.1093/ntr/ntz143.

Verplaetse, T.L., Moore, K.E., Pittman, B.P., Roberts, W., Oberleitner, L.M., Peltier, M.K.R., Hacker, R., Cosgrove, K.P., McKee, S.A., 2019. Intersection of e-cigarette use and gender on transitions in cigarette smoking status: Findings across Waves 1 and 2 of the Population Assessment of Tobacco and Health Study. Nicotine Tob. Res. 21, 10, 1423-1428. DOI:10.1093/ntr/nty187.

Watkins, S.L., Thrul, J., Max, W., Ling, P.M., 2019. Real-world effectiveness of smoking cessation strategies for young and older adults: Findings from a nationally representative cohort. Nicotine Tob. Res. 22, 9, 1560-1568. DOI:10.1093/ntr/ntz223.
